# Development of blend PEG-PES/NMP-DMF mixed matrix membrane for CO_2_/N_2_ separation

**DOI:** 10.1007/s11356-022-20168-3

**Published:** 2022-06-03

**Authors:** Ashvin Viknesh Mahenthiran, Zeinab Abbas Jawad, Bridgid Lai Fui Chin

**Affiliations:** 1 Department of Chemical and Energy Engineering, Faculty of Engineering and Science, Curtin University Malaysia, 250 CDT, 98009 Miri, Sarawak Malaysia; 2 Department of Chemical Engineering, College of Engineering, Qatar University, P.O. Box: 2713, Doha, Qatar

**Keywords:** CO_2_/N_2_ separation, Membrane technology, Mixed matrix membrane (MMM), Poly(ether sulfone) (PES), Poly(ethylene glycol) (PEG), Functionalised multi-walled carbon nanotubes (MWCNTs-F)

## Abstract

The carbon dioxide (CO_2_) separation technology has become a focus recently, and a developed example is the membrane technology. It is an alternative form of enhanced gas separation performance above the Robeson upper bound line resulting in the idea of mixed matrix membranes (MMMs). With attention given to membrane technologies, the MMMs were fabricated to have the most desirable gas separation performance. In this work, blend MMMs were synthesised by using two polymers, namely, poly(ether sulfone) (PES) and poly (ethylene glycol) (PEG). These polymers were dissolved in blend N-methyl-2-pyrrolidone (NMP) and dimethylformamide (DMF) solvents with the functionalised multi-walled carbon nanotubes (MWCNTs-F) fillers by using the mixing solution method. The embedding of the pristine MWCNTs and MWCNTs-F within the new synthesised MMM was then studied towards CO_2_/N_2_ separation. In addition, the optimisation of the loading of MWCNTs-F for blend MMM for CO_2_/N_2_ separation was also studied. The experimental results showed that the functionalised MWCNTs (MWCNTs-F) were a better choice at enhancing gas separation compared to the pristine MWCNTs (MWCNTs-P). Additionally, the effects of MWCNTs-F at loadings 0.01 to 0.05% were studied along with the polymer compositions for PES:PEG of 10:20, 20:20 and 30:10. Both these parameters of study affect the manner of gas separation performance in the blend MMMs. Overall, the best performing membrane showed a selectivity value of 1.01 + 0.05 for a blend MMM (MMM-0.03F) fabricated with 20 wt% of PES, 20 wt% of PEG and 0.03 wt% of MWCNTs-F. The MMM-0.03F was able to withstand a pressure of 2 bar, illustrating its mechanical strength and ability to be used in the post combustion carbon capture application industries where the flue gas pressure is at 1.01 bar.

## Introduction

The increase in global warming and climate change is a result of excessive green house gas (GHG) emissions in the atmosphere (Kim et al. 2020). Industrialisation and the demand for world energy from large volumes of fossil fuels have caused substantial CO_2_ pollution from power plants into the environment (Vinoba et al. 2017). Therefore, the separation of CO_2_/N_2_ is a critical problem in the industrial sector (Heo et al. 2020). Over recent years, commercial polymers of poly(ether sulfone) (PES) have been used for gas separation due to its excellent thermal and mechanical strengths as well as an ether-oxygen unit. PES can bind with CO_2_ as it has a polar backbone of higher degree of chain rigidity causing better CO_2_ selectivity (Garcia-Ivars et al. 2016; W. Choi et al. 2015; Kamal et al. 2014; Mubashir et al. 2019a, 2020). Alternatively, an additive, poly(ethylene glycol) (PEG) is a commonly studied polymer because it has strong affinity towards CO_2_ (Akbarian et al. 2018). Thus, it improves the CO_2_ permeation of polymeric membranes (Mannan et al. 2013). Blending polymers promote a higher selectivity of permeability of CO_2_ due to the PES and PEG polymers, hence synthesising a blend mixed matrix membrane (MMM) with better CO_2_/N_2_ separation (Mannan et al. 2013). Other essential parameters are the solvents used as they affect the membrane’s morphology (Isanejad et al. 2017). The use of N-methyl-2-pyrrolidone (NMP) is to improve the permeability of CO_2_ (M.S. Ahmad et al. 2018). This is because NMP reduces the non-selective voids and increases the selectivity of the gases due to the enhancement of the hydrogen bonding with the OH^−^ groups of the polymers (Mubashir et al. 2018a). On the other hand, dimethylformamide (DMF) is concluded to have close gas separation properties and solubility properties to a structure of (4,40-methylenebis(2,6-dimethyl)-2,2-bis(3,4dicarboxylphenyl)hexafluoropropane/pyromellitic) dimide copolyimide membrane, which means it has a high selectivity of CO_2_ and N_2_ (Isanejad et al. 2017; Ahmad et al. 2018). According to Ahmad et al. (2018), it showed that the PES-DMF membrane obtained a CO_2_/CH_4_ separation selectivity of 2.56 compared to the PES-NMP or PES-DMAc membranes. Additionally, DMF has a low density and viscosity compared to water, thereby allowing it to be more efficient and capable for CO_2_ solubility (Jödecke et al. 2012). Thus, the two solvents are used to promote a higher selectivity and permeability of CO_2_/N_2_ separation.

Furthermore, besides the presence of polymers, and solvents, the inorganic fillers such as Mubashir et al. 2018a(MOF) (Mubashir et al. 2019a, 2022), amine functionalised MOF with MIL-53 topology zeolite (Mubashir et al. 2016, 2018b, 2019b, 2020), zeolite (Mubashir et al. 2015, 2018a, 2018c, 2019b, 2019c) and functionalised multi-walled carbon nanotubes (MWCNTs-F) are also incorporated into the blend MMM. The MWCNTs-F is utilised as an inorganic fillers due to its excellent mechanical properties and porous structure that improve gas diffusion (Adib et al. 2015). As the MWCNTs-F is embedded into the blend MMM, it creates a strong interaction with polymer chains, which promotes a better gas diffusion behaviour (Wang et al. 2014). Furthermore, the polymers that are used act as dispersing agents to improve the MWCNTs-F dispersion uniformly throughout the blend MMM, thereby improving the gas diffusion (Zheng and Xu 2010). As a result, there is better gas diffusion of CO_2_/N_2_ leading to improved CO_2_/N_2_ separation (Aroon et al. 2010). Generally, to prevent the forming of clusters and aggregation in the nanotubes, the use of MWCNTs-F is introduced. Thus, it would create better dispersion and stability (Sanip et al. 2011). However, the full potential of the MWCNTs-F may be hindered as the optimum loading is unknown together with the blending of PES and PEG. Furthermore, the functionalisation method would also significantly increase the CNT dispersion (Goh et al. 2011). Hence, to improve the MWCNTs-F, the use of beta-cyclodextrins (β-CD) has shown some promising results (Lee et al. 2018).

There are several types of research and studies conducted on the materials of membrane fabrications. Up to date, there is no work investigating the blend MMM, which is synthesised from blending PES/PEG polymers dissolved in NMP and DMF solvents with MWCNTs-F loading to enable better CO_2_/N_2_ separation. It is expected that the polar aromatic ether (C–O) and (O–H) groupings detected in the blend membranes interrelate well with the non-polar (CO_2_). This enhances the separation towards CO_2_/N_2_. The polymers of PES/PEG together with the solvents of NMP and DMF and MWCNTs-F loading are selected for this research as their properties promote a higher selectivity and permeance of CO_2_/N_2_ separation. For the first time, this research uses a Hansen solubility parameter analysis to study the integration and compatibility of the polymers, solvents, and MWCNTs. Additionally, the research studies the effects of varying the MWCNTs-F percentage loading for CO_2_/N_2_ separation. Successful results of this project are expected to increase both the selectivity and the permeance of CO_2_/N_2_ separation. As a result, the newly synthesised blend MMM is expected to be insights in reducing the energy requirements and costs and improving the efficiency of gas separation in the industrial sector.

## Research methodology

### Chemical materials

The multi-walled carbon nanotubes (MWCNTs) with 95% purity were purchased from Shenzen (China). The PES and PEG were supplied from Aldrich, Malaysia. The N-methyl-2-pyrrolidone (NMP), dimethylformamide (DMF), acetone and ethanol were provided from Merck, Malaysia. The gases (carbon dioxide (CO_2_) and nitrogen (N_2_)) were purchased from Eastern Oxygen Industries Sdn. Bhd., Malaysia.

### MWCNTs-F

During the preparation, the drying was carried out at 120 °C to remove all the moisture for the functionalisation of MWCNTs. Thereafter, the MWCNTs were functionalised via the Chen’s soft cutting method (Lee et al. 2018; Chen et al. 2001). Next, a mortar and pestle were used to ground the pristine MWCNTs (MWCNTs-P). Simultaneously, ethanol is added to the MWCNTs-P to produce a greyish sticky mixture. Furthermore, grinding was carried out for 2.5 h and a fine grey powder was produced, which was then heated at 80 °C for 24 h. The final product was the MWCNTs-F (Lee et al. 2018).

### Fabrication of MMM

The fabrication of the MMM was carried out with the combination of the wet-phase inversion method followed by solvent exchange with solvents. This was to remove any moisture on the membrane. Next, a controlled quantity of MWCNTs was added to the solvent of PEG and sonicated for 20 min (Lee et al. 2018), as tabulated in Table 1. Finally, casting was performed by pouring the solution on a glass plate at the casting thickness of 300 μm. Lastly, the membrane was placed in an oven at 60 °C for 24 h (Akbarian et al. 2018) before being stored and used.

**Table 1:** List of membranes fabricated

Membrane	PES (wt%)	PEG (wt%)	NMP (wt%)	DMF (wt%)	MWCNTs-F (wt%)	MWCNTs-P (wt%)
MMM-0.01F	20	20	29.94	29.94	0.01	-
MMM-0.02F	20	20	29.88	29.88	0.02	-
MMM-0.03F	20	20	29.81	29.81	0.03	-
MMM-0.035F	20	20	29.78	29.78	0.035	-
MMM-0.04F	20	20	29.75	29.75	0.04	-
MMM-0.05F	20	20	29.69	29.69	0.05	-
MMM-0.03P	20	20	29.81	29.81	-	0.03
MMM-PEG30-0.03F	10	30	29.81	29.81	0.03	-
MMM-PEG10-0.03F	30	10	29.81	29.81	0.03	-

### Hansen solubility parameters

To estimate the type of interactive forces responsible for the compatibility between materials, the Hansen solubility parameter (HSP) was carried out. The HSP was based on the cohesive energy in the material where it was divided into three parts, which correspond to atomic dispersion (*δ*_*d*_), molecular dipolar interaction (*δ*_*p*_) and hydrogen-bonding interaction (*δ*_*h*_) (Andecochea et al. 2018; Lapuerta and Canoira 2016). A ternary diagram was used to graphically observe the positions of these three parts as well as to observe the relative positions of polymers, solvents and inorganic fillers. Ternary diagrams were plotted using the normalised HSP values to translate the dispersion, polar and hydrogen components into a two-dimensional graph. The graph enabled a theoretical explanation of the solubility of polymers, solvents and inorganic fillers with each other. If the points were closer to each other, the higher the solubility, the better would be the results of permeance and selectivity. In the ternary graph, the left axis represented the dispersion (*D*) of the component; the bottom axis represented the polarity (*P*) of the component; and the right axis represented the hydrogen bonding (*H*) of the components (Abbott 2020; Andecochea et al. 2018).

Values were available in the HSP database online, which were known to be the absolute HSP values. However, the plotting of a ternary diagram required normalised HSP values. As the ternary graph was plotted, the main study for HSP were the distances between materials in the graph known as relative affinity, which determines the compatibility between materials. The equations below present the calculations of relative affinity in terms of two and three components (Abbott 2020; Andecochea et al. 2018).

For two components

1 $$\mathrm{Relative}\ \mathrm{Affinity}=\overline{AB}=\sqrt{\left({\left({\delta}_{dA}-{\delta}_{dB}\right)}^2+{\left({\delta}_{pA}-{\delta}_{pB}\right)}^2+{\left({\delta}_{hA}-{\delta}_{hB}\right)}^2\right)}$$

For three components

2 $$\mathrm{Relative}\ \mathrm{Affinity}=\overline{AC}-\overline{BC}=\sqrt{\left({\left({\delta}_{dA}-{\delta}_{dC}\right)}^2+{\left({\delta}_{pA}-{\delta}_{pC}\right)}^2+{\left({\delta}_{hA}-{\delta}_{hC}\right)}^2\right)}-\sqrt{\left({\left({\delta}_{dB}-{\delta}_{dC}\right)}^2+{\left({\delta}_{pB}-{\delta}_{pC}\right)}^2+{\left({\delta}_{hB}-{\delta}_{hC}\right)}^2\right)}$$

### Permeation test for CO_2_/N_2_

A gas permeation test was conducted based on the method explained in the previous published work (Lee et al. 2018).

### Membrane characterisation study

#### ATR-FTIR

The benefits of using an attenuated total reflectance Fourier transform infrared spectroscopy (ATR-FTIR) were to understand the chemical properties of the blend MMM fabricated. The ATR-FTIR was obtained by setting a spectrometer ranging from 400 to 4000 cm^−1^. Ideally, 32 scans were collected with 4 cm^−1^ resolutions from data at room conditions. Furthermore, to ensure accuracy of the results, it was recommended to take three images of each sample.

#### Drop shot analysis (contact angle)

This membrane characterisation study was used to measure the wettability of the membrane. The contact angle of a flat surface of the membrane was measured using a single sessile drop with a telescope, which had an adjustable crosshair. These angles enabled the analysis of the properties of the membrane in terms of hydrophilicity and repulsion forces between the interfacial properties. To obtain an accurate and precise angle, 10 readings were taken for each membrane sample.

#### SEM

In this research, the technique of scanning electron microscope (SEM) was used to understand the morphology and cross-sectional structure of the synthesised blend MMMs (SEM, Hitachi TM3000, Tokyo, Japan). Prior to scanning, samples of the membranes were obtained by fracturing the membrane. This was conducted by freezing the membrane to −80 °C for 24 h to allow for a clean fracture. Additionally, platinum was coated on top of the sample to help prevent charging, thereby producing a good contrasting image. A variety of membrane samples were taken to confirm consistencies within the experimental results.

#### AFM

In this study, the mean roughness (*R*_a_) and root mean square (*R*_RMS_) of the PEG-PES/NMP-DMF mixed matrix membranes were measured using atomic force microscope MFP-3D system (Asylum Research, USA) in the scan size of 20 μm × 20 μm.

## Results and discussions

### Overview

The purpose of this research is to fabricate blend mixed matrix membranes (MMMs) using PES/PEG dissolved in NMP/DMF solvents with MWCNTs-F and test the performance for CO_2_/N_2_ separation. To accomplish this, the pristine MWCNTs and functionalised MWCNTs are embedded into the blend PES/PEG membrane, and the loadings are then optimised. Finally, membrane characterisation is carried out on all of the fabricated blend MMMs to test and evaluate the performance of the synthesised blend MMM towards a high selectivity and permeance of CO_2_/N_2_ separation. Testing and characterisations such as the HSP study, contact angle analysis, ATR-FTIR testing and gas permeation testing were all carried out to enable a comprehensive understanding of the results collected.

### Overall compatibility study of HSP on blend MMM

**Fig. 1:**
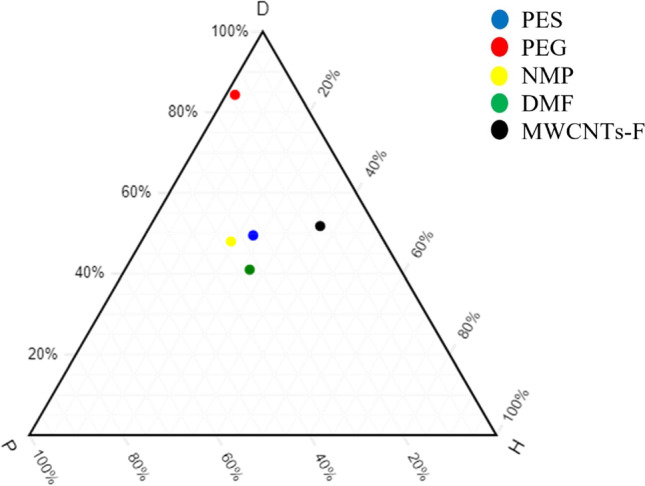
Ternary plot — PES, PEG, NMP, DMF, MWCNTs-F

A HSP method is carried out with the primary aim to understand the compatibility of blend PES/PEG polymers with blend NMP/DMF solvents and MWCNTs-F by estimating the type of interactive forces responsible for their compatibility. The HSP values for the polymers, solvents and inorganic fillers used in synthesizing the new MMMs are summarised in Table 2. Normalised calculations are also calculated to plot the HSP ternary, as seen in Fig. 1. This plot is used to demonstrate qualitatively the components that are compatible with each other, while the HSP normalised values provide a quantitative approach. In Table 2, the HSP values for PES, NMP, DMF and MWCNTs-F are closer to each other while PEG is further away. As such, the four components (PES, NMP, DMF and MWCNTs-F) are hypothesised to be closer to each other on the ternary graph, where a better gas permeation and selectivity of CO_2_/N_2_ are experienced. The calculated values are in line with the expected hypothesis, as demonstrated in Fig. 1. From the ternary graph, the normalised HSP values calculated indicate that PEG is further away from the other materials, making it harder for it to be compatible and to interact with PES, NMP, DMF and MWCNTs-F. According to Andecochea et al. (2018), the closer points to each other on the ternary graph demonstrate better interaction, which illustrates greater compatibility and greater gas permeation and selectivity (Andecochea et al. 2018). As a result, PES would have better interaction with the two solvents (NMP/DMF) and MWCNTs-F, as their relative affinity (distance of points from each other) is smaller while the HSP ternary graph significantly demonstrates that PEG has low interaction with the solvents and the MWCNTs-F. The PEG still plays a significant role in facilitating the permeation of CO_2_, due to its carbonyl group, creating an active site for the polymer matrix to interact with CO_2_ (Akbarian et al. 2018).

**Table 2:** References of absolute HSP values

Components	Absolute HSP values			Normalised			References
	δ_D_	δ_P_	δ_H_	δ_D_	δ_P_	δ_H_	
PES	19.6	10.8	9.2	49.5	27.3	23.2	Adamska and Voelkel (2006)
PEG 2000	17.7	2.9	0.4	84.3	13.8	1.9	Alvi et al. (2019)
NMP	18	12.3	7.2	48.0	32.8	19.2	Adamska and Voelkel (2006)
DMF	17.4	13.7	11.3	41.0	32.3	26.7	Andecochea et al. (2018)
MWCNTs-F	25.64	5.83	18	51.8	11.8	36.4	J. Ma et al. (2018)
MWCNTS-P	16.76	2.41	13.91	50.7	7.3	42.0

#### Compatibility study of MWCNTs with polymers

**Fig. 2:**
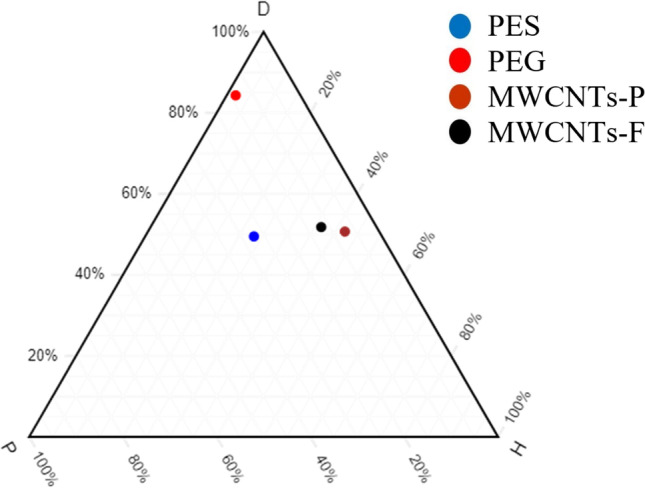
Ternary plot — MWCNTs-F and MWCNTs-P against PES/PEG

The compatibility of fabricating MMMs using MWCNTs-F and MWCNTs-P was investigated to determine the better inorganic filler. This is studied by synthesising blend MMMs that are composed of PES:PEG at a weight ratio of 20:20 and filler loading of 0.03 wt%. The normalised HSP values are plotted, as shown in Fig. 2, where MWCNTs-F is at a closer distance compared to MWCNTs-P. Hence, MWCNTs-F has better integration and compatibility with the blend PES/PEG polymers. The MWCNTs-P may have less integration with the polymers because of the agglomeration due to their stronger π–π bonds (Khan et al. 2012). This leads to a lower pore accessibility causing the CO_2_ permeance to be low. Additionally, due to entanglement because of the high ratio of length to diameter of MWCNTs-P, a lower permeance of CO_2_ is experienced. On the other hand, the MWCNTs-F has better integration due to Chen’s soft cutting method that functionalises the CNTs. The β-CD used in the cutting method shortened the MWCNTs, thus preventing entanglement and promoting dispersibility (Ahmad et al. 2014).

#### Compatibility study of MWCNTs with solvents

**Fig. 3:**
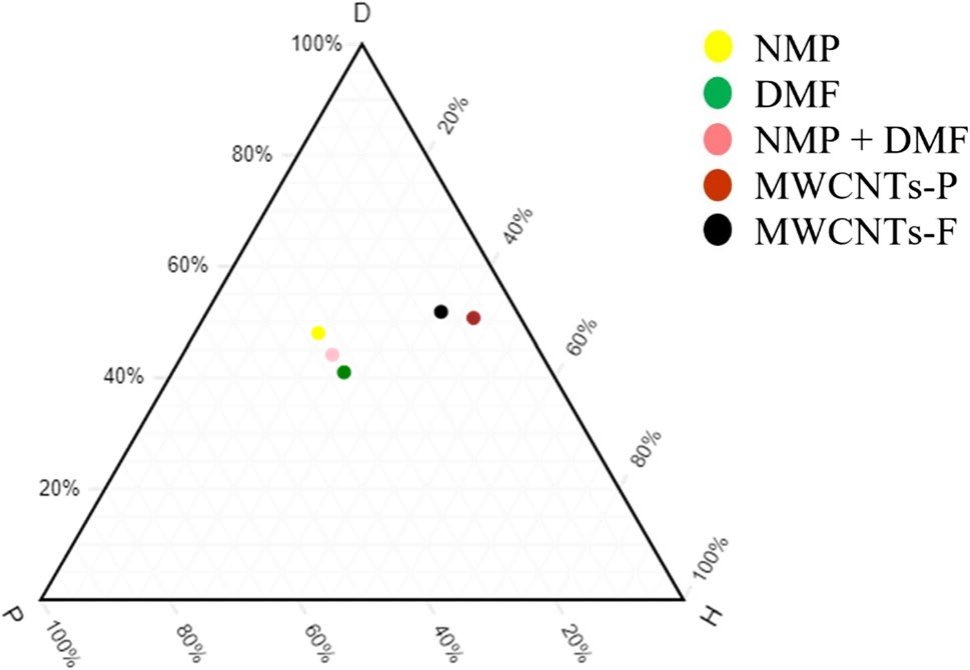
Ternary plot — MWCNTs-F and MWCNTs-P against NMP/DMF

For the next study, the integration of MWCNTs-F and MWCNTs-P is compared with NMP, NMF and NMP and DMF. This study indicates the type of MWCNT filler that is compatible with the two types of solvents or a combination of the solvents. As seen from Fig. 3, MWCNTs-F is closer to all three variations of the solvents. However, the solvent combination of NMP and DMF along with MWCNTs-F has the closest attraction and, as such, can be concluded as the ideal solvent and filler choice.

#### Compatibility study of polymer composition with MWCNTs-F and solvents

**Fig. 4:**
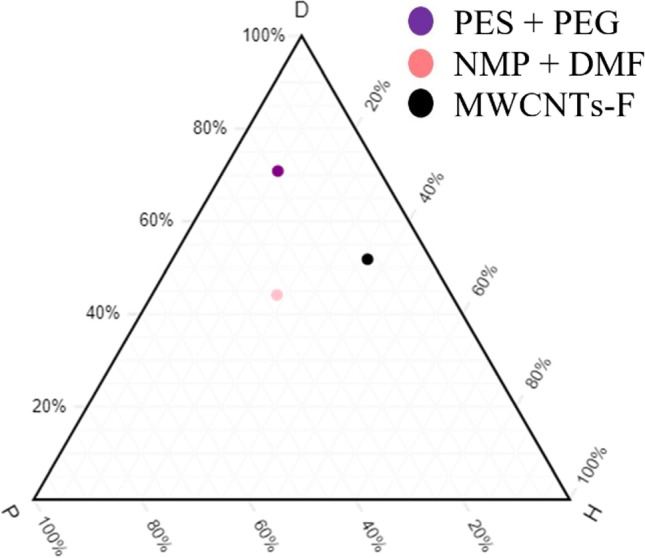
Polymer ratio (10:30, PES:PEG)

**Fig. 5:**
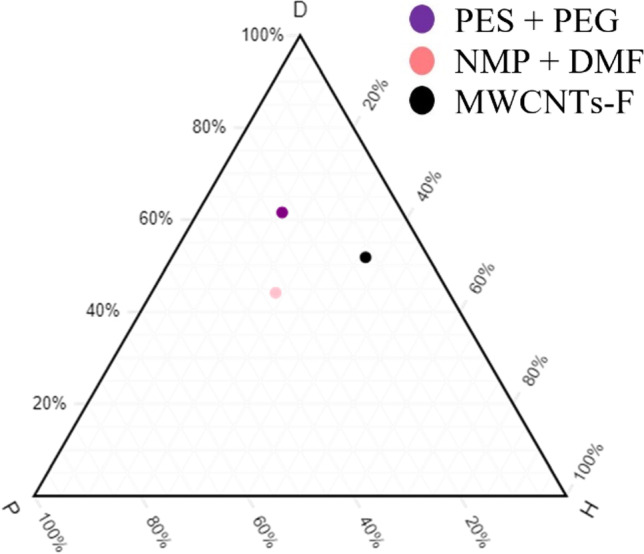
Polymer ratio (20:20, PES:PEG)

**Fig. 6:**
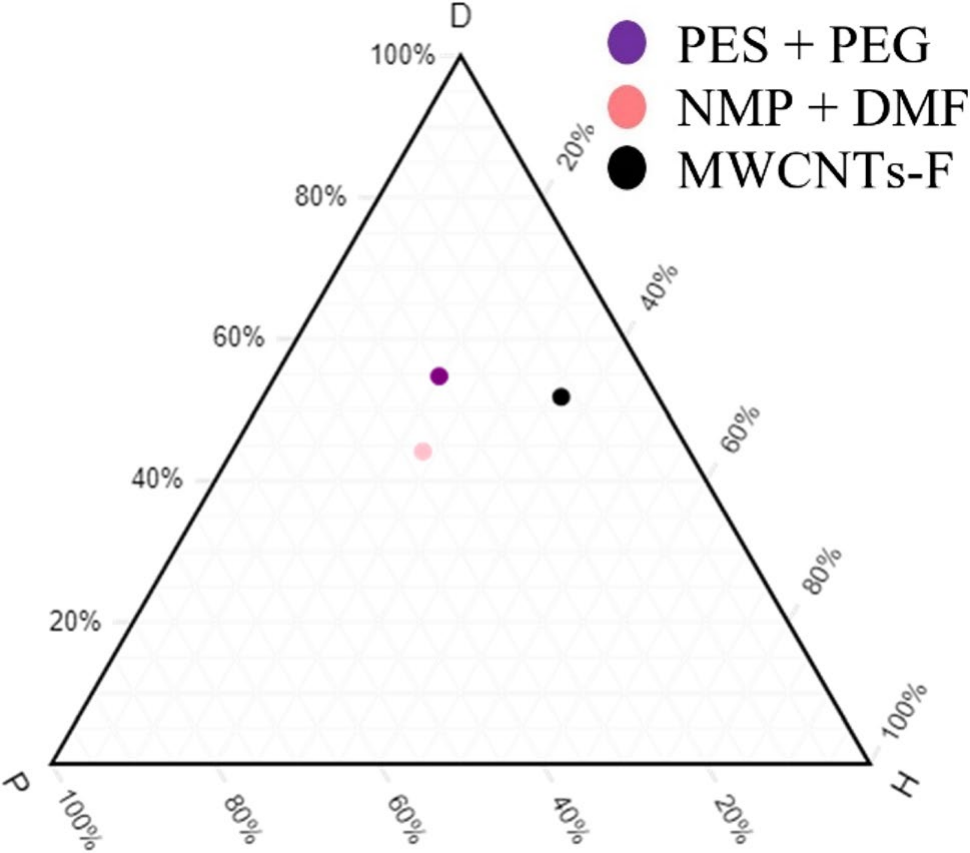
Polymer ratio (30:10, PES:PEG)

Following the choice of inorganic filler, the optimum polymer composition is to be studied. As shown in Fig. 4, a polymer weight ratio of 10 PES and 30 PEG is investigated. In Figs. 5 and 6, weight ratios of 20 PES and 20 PEG and 30 PES and 10 PEG are described, respectively. As observed from the three ternary diagrams below, the plots on Fig. 6 are closer to one another when compared to Figs. 4 and 5. As Fig. 6 has a higher ratio of PES to PEG, it resulted in better interaction among component, gas permeation and selectivity. However, this is not necessarily true as the increase in PES can cause an intermixing of ether linkages with the PEG, which results in a decrease of the sorption sites. As such, Fig. 5 representing an equal ratio of PES and PEG may be ideal as it allows for an appropriate increase in PEG to help improve the CO_2_ permeance as well as the polar ether groups that have an affinity to CO_2_ molecules. However, the ratio of 10:30 PES and PEG in Fig. 4 weakens the mechanical strength of the MMMs as the PES ratio is lower. Hence, the hypothesis is strengthened that blend MMM weight ratio of 20 PES and 20 PEG is ideal. Looking at the relative affinity (distance in the graph) values, Fig. 4 has a relative affinity value of 7.5, Fig. 5 has a value of 0.5 and Fig. 6 has a value of 4.3. The smaller the values, the better the compatibility and interaction. Thus, this hypothesis investigates was further investigated by studying the optimum loading of MWCNTs-F through gas permeation, ATR-FTIR and contact angle studies.

### Effect of integrating MWCNTs in a PES-PEG blend membrane

#### CO_2_ and N_2_ separation performance

**Fig. 7:**
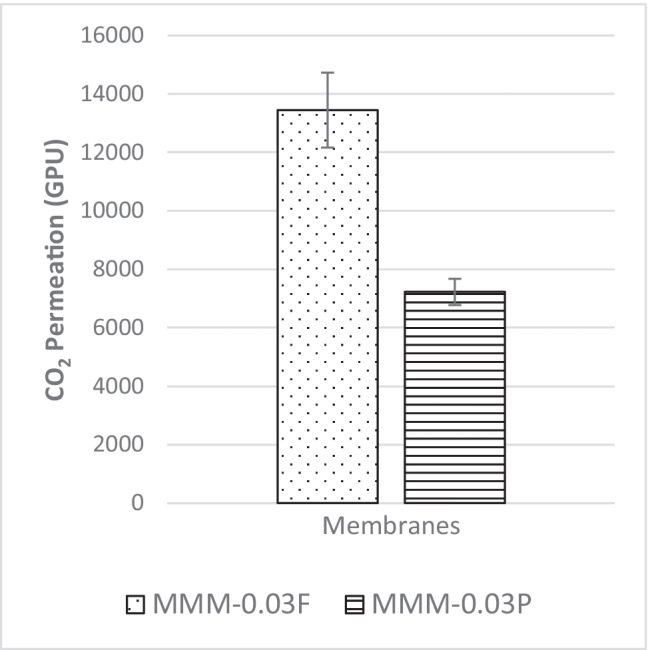
CO_2_ permeance of PES-PEG blend membrane, MMM-0.03F and MMM-0.03P, prepared with 0.03 wt% of functionali**s**ed and pristine MWCNTs

**Fig. 8:**
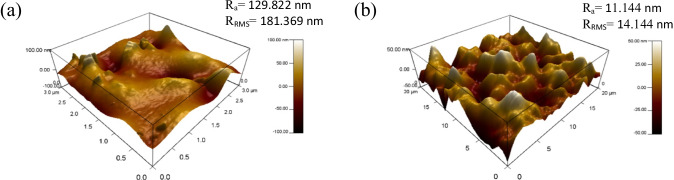
AFM morphologies of the mixed matrix membrane surface for **a** MMM-0.03P and **b** MMM-0.03F

An investigation into the effect of MWCNTs within blend MMMs of PES/PEG is studied through a single gas permeation test of CO_2_ and N_2_. Figure 7 illustrates the CO_2_ permeance of the two fabricated MMMs. MMM-0.03P is synthesised by blending the weight ratios of 20 PES and 20 PEG with NMP/DMF solvents and MWCNTs-P loading of 0.03 wt%. This results in a CO_2_ permeance of 7225.6 GPU. Meanwhile, MMM-0.03F is fabricated by blending the weight ratio of 20 PES and 20 PEG with NMP/DMF solvents and MWCNTs-F loading of 0.03 wt%. It shows a CO_2_ permeance of 13,441.17 GPU. The results show that a decrease of CO_2_ permeance from MMM-0.03F to MMM-0.03P is due to agglomeration (Aroon et al. 2010). The agglomeration in MMM-0.03P is because of the MWCNTs-P having stronger π–π bonds (Khan et al. 2012) which caused a rough surface with large mean roughness (*R*_a_) and root mean square (*R*_RMS_) values (Fig. 8a) as compared to MMM-0.03F (Fig. 8b). Furthermore, agglomeration hinders gas transportation due to reduced pore sizes. As pore accessibility is smaller, it results in the gas transportation being slower, thus reducing the gas movement (Wong et al. 2018). In addition, the MMM-0.03P shows a lower CO_2_ permeance due to entanglement, which is the result of a high ratio of length to the diameter of MWCNTs-P contributing to agglomeration (Wong et al. 2018).

The MMM-0.03F has a higher CO_2_ permeance of 13,441.17 GPU and shows the best gas separation among all the membranes. Due to the method of functionalisation, the MWCNTs-F has a better gas separation property. By using Chen’s soft cutting method, the MWCNTs-F is made more dispersible within the blend MMM (Ahmad et al. 2014). As the soft cutting method is done with β-CD, this shortens the MWCNTs, thereby preventing entanglement and promoting dispersibility. A study by Ahmad et al. (2014) states that the dispersibility of MWCNTs improve because of the interactions between the hydrogen bonding, van der Waals forces and the β-CD coating on the MWCNTs-F, thus creating a repelling behaviour that results in an enhanced dispersibility in the blend MMM (A.L. Ahmad et al. 2014). In addition, entanglement is minimised due to the method of functionalisation, as the MWCNTs-F disperses in the blend MMM without any agglomeration. This results in a better movement of the CO_2_ molecules, hence providing an improved CO_2_ permeance (Wong et al. 2018).

**Fig. 9:**
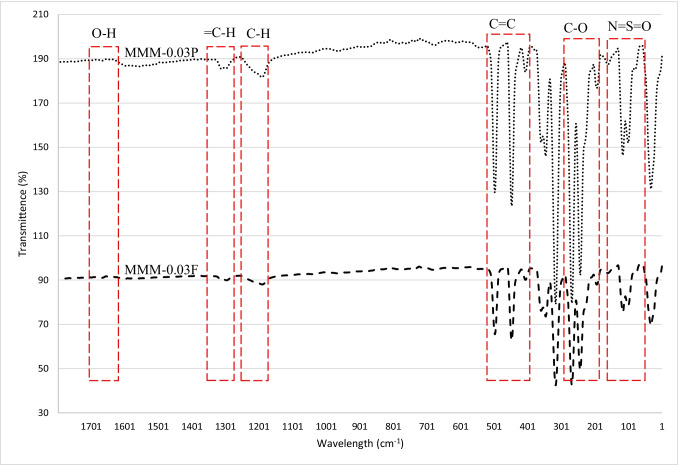
FTIR results of PES-PEG blend membrane, MMM-0.03F and MMM-0.03P, prepared with 0.03 wt% of functionalised and Pristine MWCNTs

The FTIR study carried out in Fig. 9 illustrates the highest peak as O–H at 3635.29 cm^−1^, resulting in an increase in dipole interaction between O–H and CO_2_ molecules that allows higher CO_2_ permeance. Based on Fig. 7, the MMM-0.03F demonstrates higher CO_2_ permeance, indicating that the O–H groups present are larger as compared to MMM-0.03P.

**Fig. 10:**
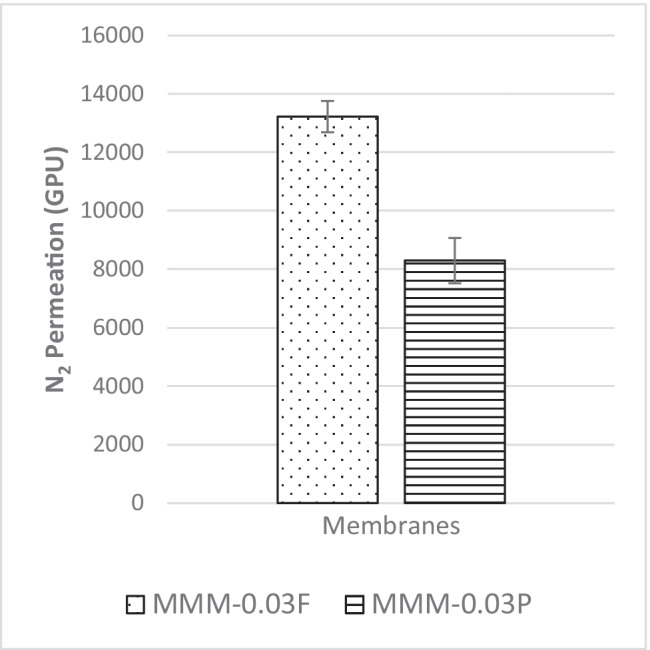
N_2_ permeance of PES-PEG blend membrane, MMM-0.03F and MMM-0.03P, prepared with 0.03 wt% of functionalised and pristine MWCNTs

In Fig. 10, the results of the N_2_ permeance for the membranes MMM-0.03F and MMM-0.03P are plotted. Figure 10 indicates that MMM-0.03F has better N_2_ permeance of 13,229.30 GPU compared with MMM-0.03P, which has N_2_ permeance of 8294.13 GPU. The MMM-0.03P permeance is lower compared to MMM-0.03F because of the agglomeration occurring in the membrane structure as the pores are smaller, resulting in higher resistance to permeate N_2_ (Wong et al. 2018). On the other hand, MMM-0.03F has higher N_2_ permeance because of the soft cutting method performed during functionalisation. This is similar to the CO_2_ permeance. However, according to Lee et al. (2018), there is some resistance for the N_2_ permeance as demonstrated in MMM-0.03F because MWCNTs-F favours less N_2_ molecules (Ahmad et al. 2014; Lee et al. 2018).

**Fig. 11:**
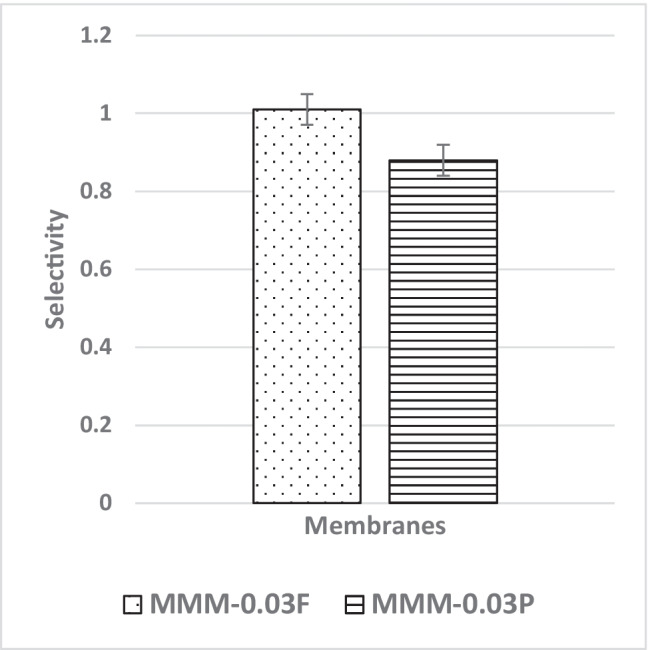
CO_2_/N_2_ selectivity of PES-PEG blend membrane, MMM-0.03F and MMM-0.03P, prepared with 0.03 wt% of functionalised and pristine MWCNTs

**Fig. 12:**
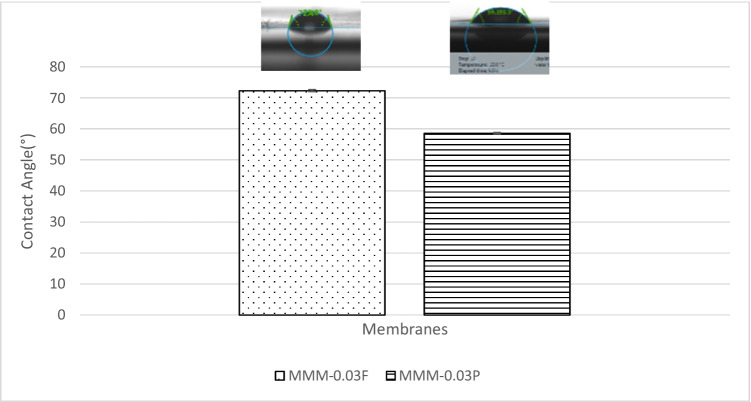
Contact angle of PES-PEG blend membrane, MMM-0.03F and MMM-0.03P, prepared with 0.03 wt% of functionalised and pristine MWCNTs

The CO_2_/N_2_ selectivity of MMM-0.03F and MMM-0.03P is displayed in Fig. 11. The selectivity for MMM-0.03F and MMM-0.03P is 1.01 and 0.879, respectively. The MMM-0.03P shows a lower selectivity due to the agglomeration experienced in the CO_2_ and N_2_ permeance. This is further confirmed by the contact angle study (Fig. 12), where the hydrophilicity of the two membranes is characterised. The MMM-0.03P has a lower contact angle of 58.64°, whereas MMM-0.03F has a contact angle of 72.4°, indicating that MMM-0.03P should have a higher selectivity as the polar functional groups (C–O and O–H) are reacting with non-polar CO_2_ (Lee et al. 2018). However, due to the agglomeration experienced in MMM-0.03P, it does not have a higher selectivity compared to MMM-0.03F. Additionally, MWCNTs-P has a higher absorption capacity of N_2_ (8294.13 GPU) as compared to CO_2_ permeance (7225.6 GPU). Thus, it reduces the CO_2_/N_2_ selectivity (Khan et al. 2012). The MMM-0.03F has a higher selectivity due to its compatibility between MWCNTs, β-CD and polymer matrix as stated by Lee et al. (2018) and as concluded in the HSP study (Fig. 2), where the relative affinity is small (Lee et al. 2018). Furthermore, in MMM-0.03F, the β-CD within the MWCNTs-F captures the CO_2_ molecules resulting in an increase of selectivity (A.L. Ahmad et al. 2014). Upon comparison of the relative affinity values for MMM-0.03P and MMM-0.03F, it indicates that MMM-0.03F has a smaller relative value resulting in better compatibility and interaction with PES/PEG and NMP/DMF, thereby making MWCNTs-F as the optimum choice between the two inorganic fillers.

**Fig. 13:**
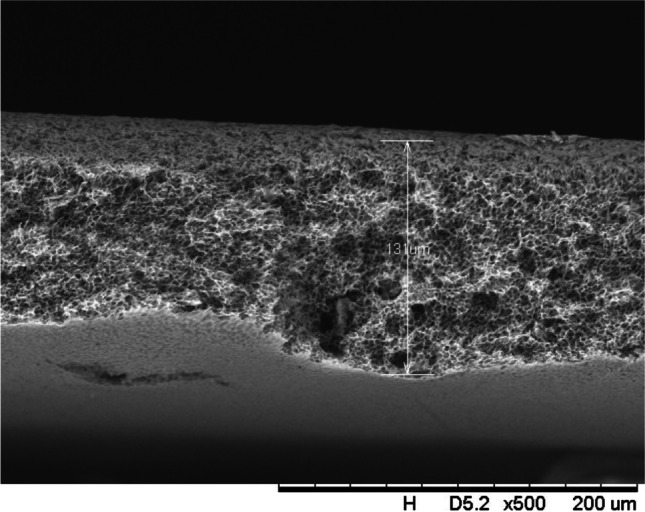
Cross section of PES-PEG blend membrane, MMM-0.03P, prepared with 0.03 wt% of pristine MWCNTs

**Fig. 14:**
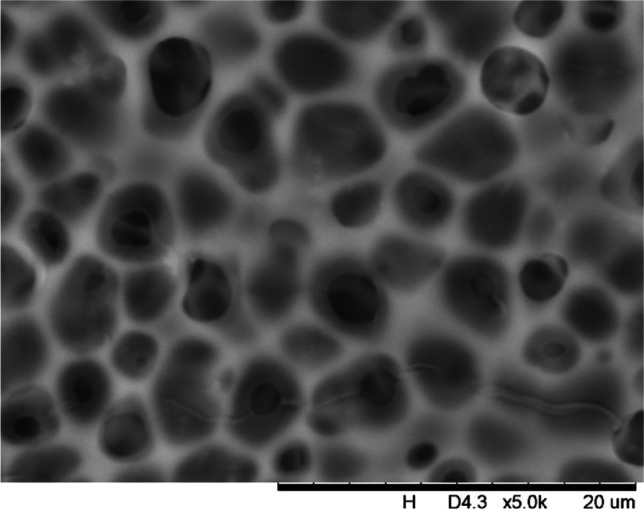
Surface of PES-PEG blend membrane, MMM-0.03P, prepared with 0.03 wt% of Pristine MWCNTs

**Fig. 15:**
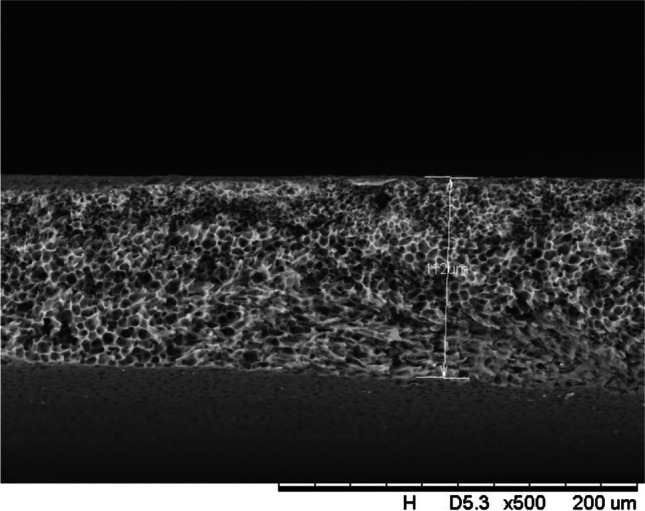
Cross section of PES-PEG blend membrane, MMM-0.03F, prepared with 0.03 wt% of functionalised MWCNTs

**Fig. 16:**
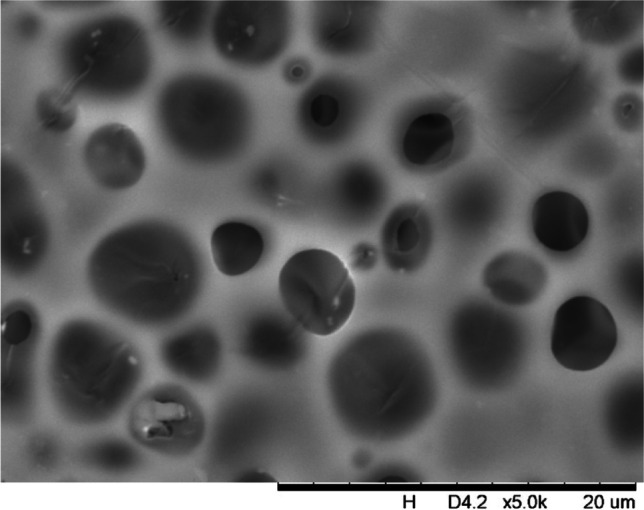
Surface of PES-PEG blend membrane, MMM-0.03F, prepared with 0.03 wt% of functionalised MWCNTs

In addition, a scanning electron microscope (SEM) characterisation is carried out illustrating the blend MMM morphology. The following Figs. 13 and 14 indicate the cross-sectional and surface image of MMM-0.03P, respectively. From these two images, there is an agglomeration in the dense sponge-like membrane due to stronger π–π bonds among the aggregated MWCNTs-P and the hindered pore sizes, where the average pore size is 1.8 + 0.18 μm (Ahmad et al. 2014). Meanwhile, the SEM analysis for MMM-0.03F is represented by Figs. 15 and 16, where the pore sizes are an average of 3.35 + 0.29 μm. The SEM results showcase that MMM-0.03F has a denser sponge-like membrane throughout the structure. In overall, this denser structure might be due to the hydrophilic MWCNTs which suppress the overall diffusion and delayed the exchange between solvent and non-solvent during the phase inversion (Choi et al. 2006; Ahmad et al. 2014).

### Effect of polymer composition

#### CO_2_ and N_2_ separation performance

**Fig. 17:**
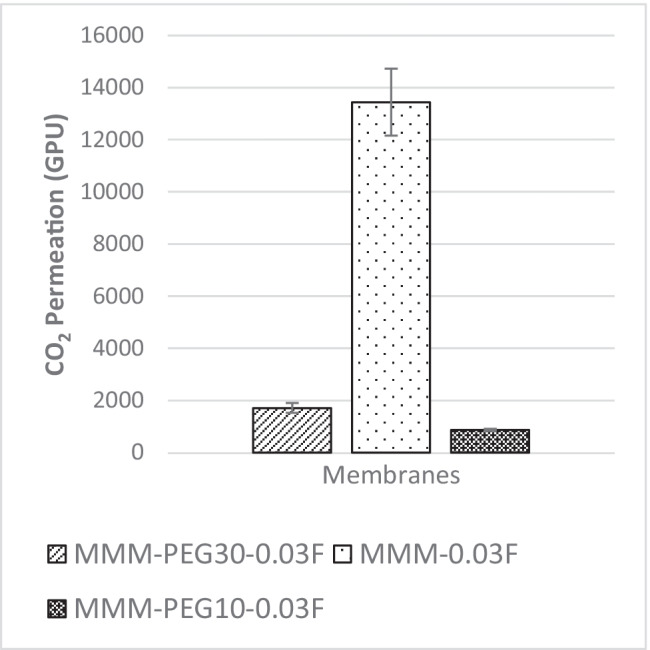
CO_2_ permeance of PES-PEG blend membrane, MMM-PEG30-0.03F, MMM-0.03F and MMM-PEG10-0.03F, prepared at different polymer compositions of 30 wt%, 20 wt% and 10 wt% of PEG

**Fig. 18:**
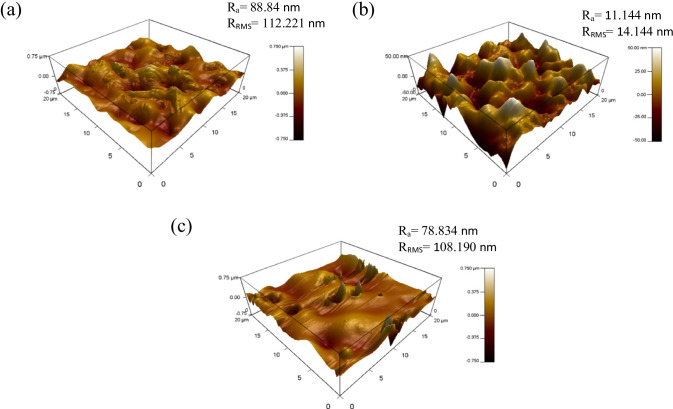
AFM morphologies of the mixed matrix membrane surface for **a** MMM-PEG30-0.03F, **b** MMM-0.03F and **c** MMM-PEG10-0.03F

**Fig. 19:**
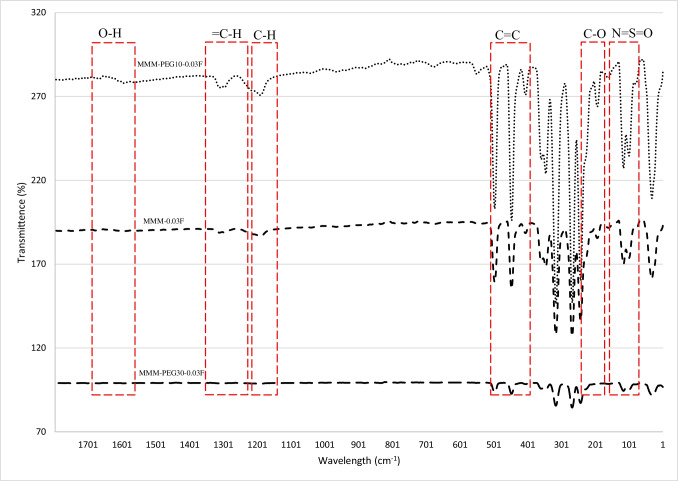
FTIR results PES-PEG blend membrane, MMM-PEG30-0.03F, MMM-0.03F and MMM-PEG10-0.03F, prepared at different polymer compositions of 30 wt%, 20 wt% and 10 wt% of PEG

**Fig. 20:**
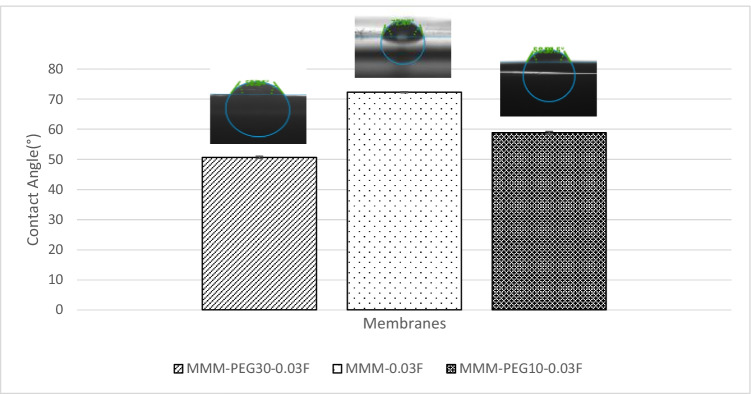
Contact angle of PES-PEG blend membrane, MMM-PEG30-0.03F, MMM-0.03F and MMM-PEG10-0.03F, prepared at different polymer compositions of 30 wt%, 20 wt% and 10 wt% of PEG

Previous works have concluded that blend MMMs with functionalised MWCNTs as the inorganic filler would be optimum for gas separation performance. As such, the following study investigates the effect of polymer composition through gas separation of blend MMMs (MMM-PEG30-0.03F, MMM-0.03F and MMM-PEG10-0.03F), by varying the PES:PEG weight ratios to 10:30, 20:20 and 30:10, respectively, with an MWCNTs-F loading of 0.03 wt%. Figure 17 shows that MMM-PEG30-0.03F, MMM-0.03F and MMM-PEG10-0.03F have CO_2_ permeance of 1704.14 GPU, 13,441.17 GPU and 869.42 GPU, respectively. The MMM-0.03F shows the best CO_2_ permeance and smooth surface with lower *R*_a_ and *R*_RMS_ (Fig. 18) as compared to MMM-PEG30-0.03F and MMM-PEG10-0.03F. According to Akbarian et al. (2018), by increasing the PEG, the permeance improves due to the increase in the polar ether group as it has an affinity to CO_2_ molecules (Akbarian et al. 2018). However, MMM-PEG30-0.03F shows that by increasing the PEG ratio, the mechanical strength weakens and, by doing so, the minimum pressure that the membrane functions is at below 0.5 bar. Hence, MMM-PEG-0.03F does not have a CO_2_ permeance greater than 5000 GPU, which is the working permeance. This can also be explained via relative affinity identified in the HSP study. Figure 4 indicates that PEG is distanced from PES, NMP/DMF and MWCNTs-F, which would make it less compatible and less interactive as the relative affinity value is 7.5 compared to MMM-0.03F where the relative affinity value is 0.75. Additionally, when compared to MMM-PEG10-0.03F, which has a weight ratio of 30 PES and 10 PEG, again it shows that the CO_2_ permeance is lower than 5000 GPU. This is because both the PEG and the PES chains are intermixed as they both have ether linkages, which in turn decreases the Langmuir sorption sites in the PES causing a decrease in the CO_2_ permeance (Ma et al. 2014; Zhang et al. 2018). The MMM-0.03F has a better CO_2_ permeance compared to MMM-PEG10-0.03F because of the weight ratio of PEG. As MMM-0.03F has a weight ratio of 20 PEG compared to MMM-PEG10-0.03F, which has a weight ratio of 10 PEG, more PEG leads to more CO_2_ sorption sites. The increase in sorption sites can be observed in Figs. 19 and 20 as the FTIR results indicates a higher O–H absorption peak and hydrophilicity. Thus, as illustrated in Fig. 17, the MMM-0.03F with its 20:20 weight ratio would be ideal for CO_2_ permeance.

**Fig. 21:**
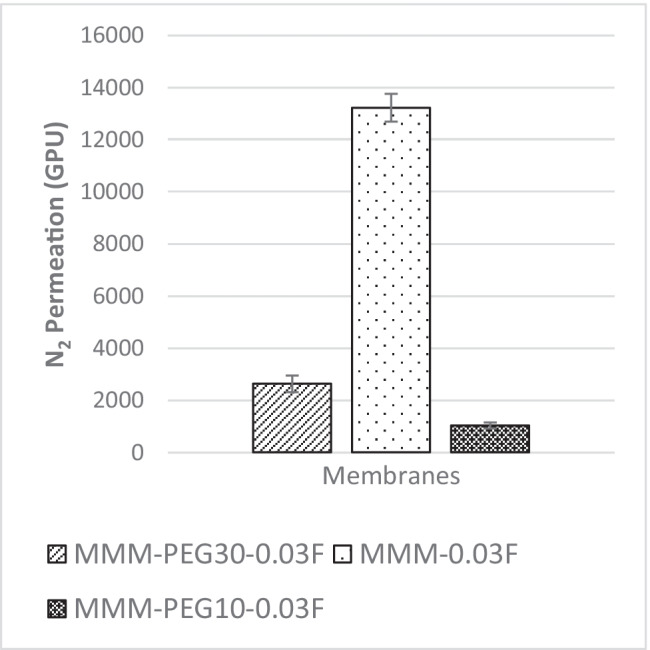
N_2_ permeance of PES-PEG blend membrane, MMM-PEG30-0.03F, MMM-0.03F and MMM-PEG10-0.03F, prepared at different polymer compositions of 30 wt%, 20 wt% and 10 wt% of PEG

On the other hand, Fig. 21 illustrates the gas separation study for N_2_ permeance. The MMM-0.03F is observed to be the best among the three membranes. The MMM-PEG30-0.03F, MMM-0.03F and MMM-PEG10-0.03F have N_2_ permeance values of 2641.58 GPU, 13,229.3 GPU and 1039.57 GPU, respectively. Both MMM-PEG30-0.03F and MMM-PEG10-0.03F indicate an inability to work at permeance of 5000 GPU, hence working permeance making MMM-0.03F with the weight ratio of 20:20 to be the best again. Upon comparison between MMM-PEG30-0.03F and MMM-PEG10-0.03F, the higher N_2_ permeance is illustrated in MMM-PEG30-0.03F because it has a higher PEG composition.

**Fig. 22:**
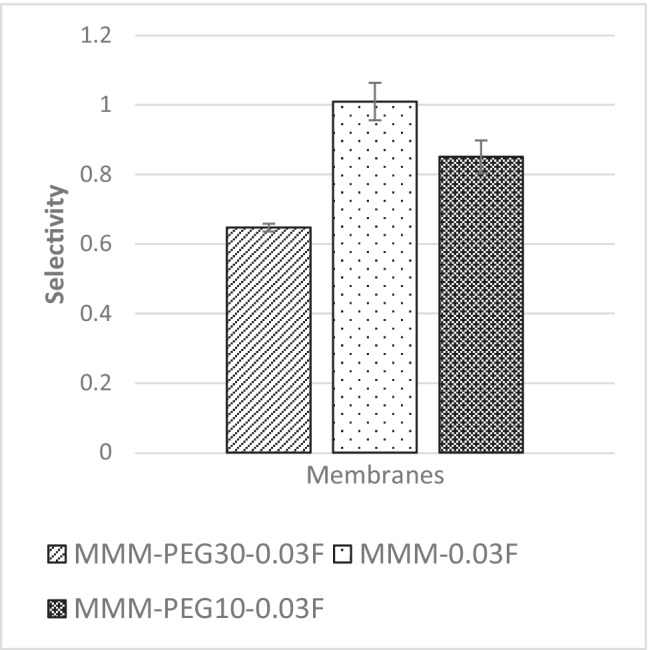
CO_2_/N_2_ selectivity of PES-PEG blend membrane, MMM-PEG30-0.03F, MMM-0.03F and MMM-PEG10-0.03F, prepared at different polymer compositions of 30 wt%, 20 wt% and 10 wt% of PEG

Figure 22 shows the CO_2_/N_2_ selectivity where MMM-PEG30-0.03F, MMM-0.03F and MMM-PEG10-0.03F have selectivity values of 0.65, 1.01 and 0.85, respectively. According to Akbarian et al. (2018), the increased PEG would lead to improved selectivity because there is an increase in sorption sites, which means more CO_2_ can be collected as it creates a stronger interaction between CO_2_ and PEG. However, MMM-0.03F has a higher selectivity due to increase in PES, which shifted the membrane towards a rubbery behaviour resulting in an increase in selectivity (Akbarian et al. 2018).

### Effect of different MWCNTs-F loading

#### CO_2_ and N_2_ separation performance

**Fig. 23:**
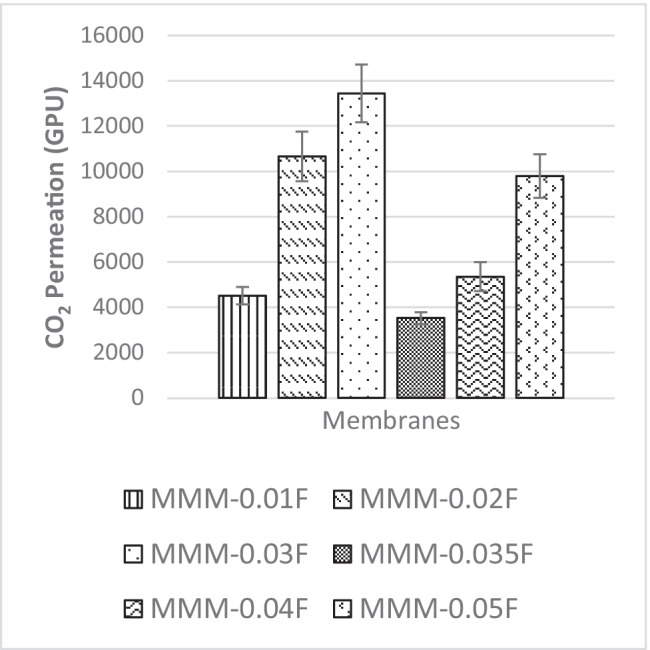
CO_2_ permeance of MMM-0.01F, MMM-0.02F, MMM-0.03F, MMM-0.035F, MMM-0.04F and MMsM-0.05F, with different MWCNTs-F loadings of 0.01 wt%, 0.02 wt%, 0.03 wt%, 0.035 wt%, 0.04 wt% and 0.05 wt%

The following study, a single gas permeation test of CO_2_ and N_2_, investigates the effects of MWCNTs-F loading within PES:PEG weight ratio of 20:20 matrix on the gas separation performance of membranes MMM-0.01F (0.01% MWCNTs-F), MMM-0.02F (0.02% MWCNTs-F), MMM-0.03F (0.03% MWCNTs-F), MMM-0.035F (0.035% MWCNTs-F), MMM-0.04F (0.04% MWCNTs-F) and MMM-0.05F (0.05% MWCNTs-F). Figure 23 demonstrates the different CO_2_ permeance at different loadings of MWCNTs-F. It shows that MMM-0.03F has the best CO_2_ permeance value of 13,441.17 GPU. The MMM-0.01F, MMM-0.02F, MMM-0.035, MMM-0.04F and MMM-0.05F demonstrated CO_2_ permeance of 4506.14 GPU, 10,654.6 GPU, 3520.92 GPU, 5348.3 GPU and 9786.03 GPU, respectively. The concept of polymer chain rigidification explains the reduction in gas permeance at a lower weight loading (Ismail et al. 2011). When the polymer chain segment is limited, a polymer chain rigidification occurs. This is due to the absorption of the polymer chain onto the surface of the fillers (Ismail et al. 2011). In MMM-0.01F, MMM-0.02F and MMM-0.03F, an increase in CO_2_ gas permeance is experienced due to the MWCNTs-F outweighing the effects of the rigid polymer chains when the loading is increased. As such, MMM-0.03F experiences high CO_2_ permeance, hence allowing for more rapid gas transportation.

**Fig. 24:**
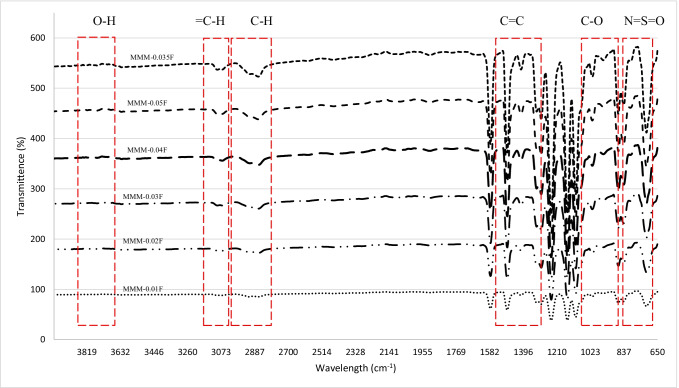
FTIR results of MMM-0.01F, MMM-0.02F, MMM-0.03F, MMM-0.035F, MMM-0.04F and MMM-0.05F, with different MWCNTs-F loadings of 0.01 wt%, 0.02 wt%, 0.03 wt%, 0.035 wt%, 0.04 wt% and 0.05 wt%

An FTIR analysis was carried out for the MMMs to investigate the functional groups within the blend MMMs. Figure 24 represents the transmittance spectra obtained for each membrane. The highest peak for the MMMs showing the strongest absorption is at 3635.29 cm^−1^, representing the O–H stretching bond. This indicates that there is a larger presence of O–H groups in the membranes resulting in an increase in dipole interaction between the O–H group and the CO_2_ molecules where a higher CO_2_ permeance is found. This is illustrated in Fig. 23 where MMM-0.03F shows a higher CO_2_ permeance, which indicates that the O–H groups present are higher compared to the other membranes.

**Fig. 25:**
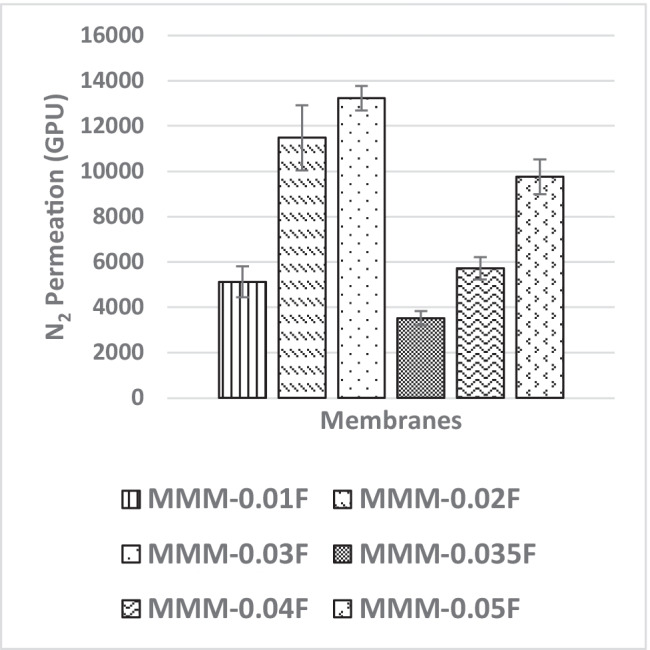
N_2_ permeance of MMM-0.01F, MMM-0.02F, MMM-0.03F, MMM-0.035F, MMM-0.04F and MMM-0.05F, with different MWCNTs-F loadings of 0.01 wt%, 0.02 wt%, 0.03 wt%, 0.035 wt%, 0.04 wt% and 0.05 wt%

The effect of different CNTs loading on N_2_ permeance is shown in Fig. 25. The MMM-0.01F, MMM-0.02F, MMM-0.03F, MMM-0.035F, MMM-0.04F and MMM-0.05F have permanence of 5121.9 GPU, 11,486.201 GPU, 13,229.30 GPU, 3508.19 GPU, MMM-0.04F 5719.21 GPU and 9760.37 GPU, respectively. A possible reason for the lower permeance at lower loadings could be due to the limited chain mobility (Ismail et al. 2011). Both MMM-0.01F and MMM-0.035F illustrate low gas permeance that could be due to the limiting effect of non-covalent MWCNTs-F, which reduces the absorption of N_2_ molecules. However, high N_2_ permeance is observed in MMM-0.02F and MMM-0.03F due to the higher MWCNTs-F loading. This improves the rapid gas transport effect and offsets the effect caused by reducing the polymer chain in N_2_ permeance. Additionally, the FTIR results, in Fig. 24, indicate that the strongest absorption peaks are at 3073.19 cm^−1^ and 2700.46 cm^−1^ representing the stretching aromatic rings of =C–H and –C–H, respectively. Next, the detection of C=C and C–O stretching bonds are at 1582.25 cm^−1^ and 1209.52 cm^−1^, respectively. Lastly, N=S=O stretching bond is at 1116.34 cm^−1^. These peaks formulate the membrane properties as either more hydrophilic or less hydrophilic. The higher the hydrophilicity is, the larger the presence of C–O grouping within the membrane. The C–O group leads to stronger intermolecular force between it and the N_2_ molecules. Hence, as shown in Fig. 25, MMM-0.02F and MMM-0.03F have higher N_2_ permeance values which could be explained from their higher C–O absorption peaks.

**Fig. 26:**
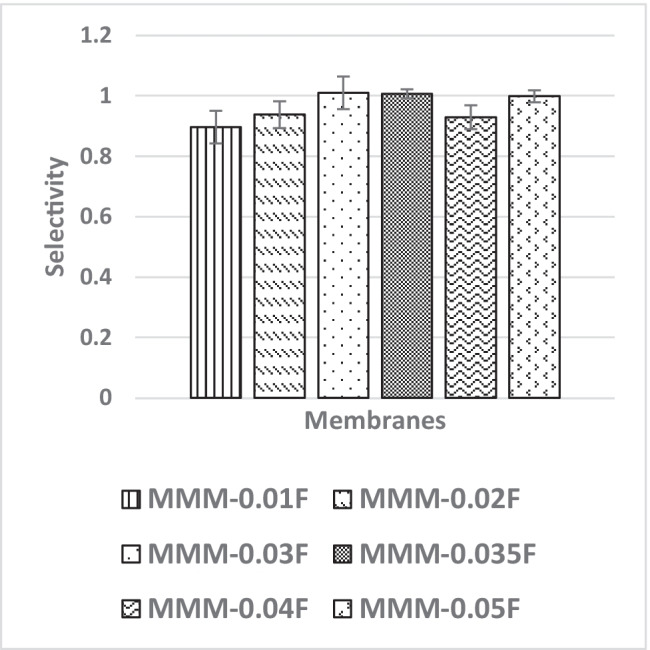
CO_2_/N_2_ selectivity of MMM-0.01F, MMM-0.02F, MMM-0.03F, MMM-0.035F, MMM-0.04F and MMM-0.05F, with different MWCNTs-F loadings of 0.01 wt%, 0.02 wt%, 0.03 wt%, 0.035 wt%, 0.04 wt% and 0.05 wt%

**Fig. 27:**
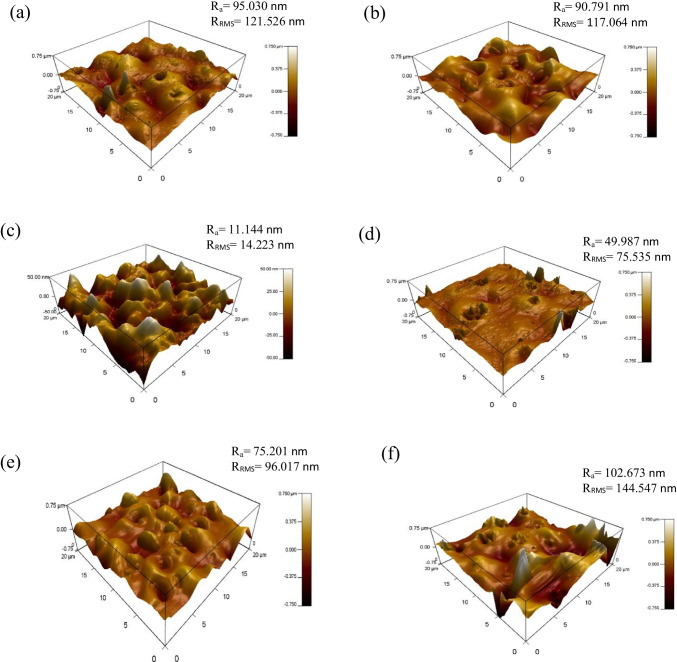
AFM morphologies of the mixed matrix membrane surface for **a** MMM-0.01F, **b** MMM-0.02F, **c** MMM-0.03F, **d** MMM-0.035F, **e** MMM-0.04F and **f** MMM-0.05F

In Fig. 26, the effect of the MWCNTs-F loadings on CO_2_/N_2_ selectivity is observed. The MMM-0.01F, MMM-0.02F, MMM-0.03F, MMM-0.035, MMM-0.04F and MMM-0.05F show selectivity of 0.897, 0.938, 1.01, 1, 0.929 and 0.999, respectively. Figure 26 shows that MMM-0.03F has the highest selectivity followed by MMM-0.035 with a close performance of 0.035 wt%. This is due to the increase in MWCNTs-F loading that provides more diffusion pathways, thus improving the difference of smaller CO_2_ molecules and larger N_2_ molecules resulting in increased selectivity. However, MMM-0.03F demonstrates the best selectivity followed by a small decrease in CO_2_/N_2_ performance for MMM-0.04F and MMM-0.05F. This is due to a reduction in the dense top layer and improves the surface roughness at the controlled loading of MWCNTs (Fig. 27).

**Fig. 28:**
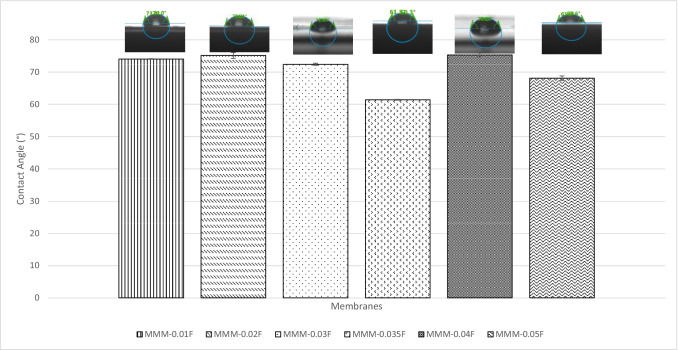
Contact angle of MMM-0.01F, MMM-0.02F, MMM-0.03F, MMM-0.035F, MMM-0.04F and MMM-0.05F, with different MWCNTs-F loadings of 0.01 wt%, 0.02 wt%, 0.03 wt%, 0.035 wt%, 0.04 wt% and 0.05 wt%

Following the single gas permeation test study and the FTIR, a contact angle analysis is conducted to investigate the wetting of the membrane. The membrane is more hydrophilic when it has a lower contact angle, making it likely to have a strong CO_2_ selectivity. Figure 28 illustrates the contact angles of membranes MMM-0.01F, MMM-0.02F, MMM-0.03F, MMM-0.035F, MMM-0.04F and MMM-0.05F at contact angles of 74.04°, 75.13°, 72.4°, 61.4°, 75.28° and 68.11°, respectively. As observed from Fig. 28*,* MMM-0.03F, MMM-0.035F and MMM-0.05F demonstrate the smallest contact angles. This indicates that these membranes particularly have a higher hydrophilic surface compared to the other membranes. Having a higher hydrophilic surface means having more C–O and O–H bonds within the polymer matrix causing a higher CO_2_ permeation and resulting in a higher CO_2_ selectivity, as illustrated in Fig. 24. Fig. 24 shows that the bonds and their transmittance are obviously at C–O and O–H. Meanwhile, based on Fig. 26, the CO_2_/N_2_ selectivity of MMM-0.03F, MMM-0.035F and MMM-0.05F demonstrates higher selectivity compared to the other membranes. This is due to its higher hydrophilic surface (Xiao et al. 2015; Suleman et al. 2018).

**Fig. 29:**
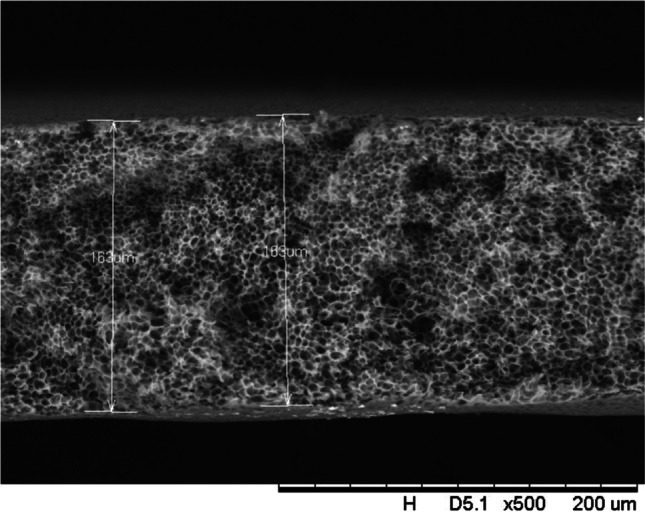
Cross section of PES-PEG blend membrane, MMM-0.01F, prepared with 0.01 wt% of functionalised MWCNTs

**Fig. 30:**
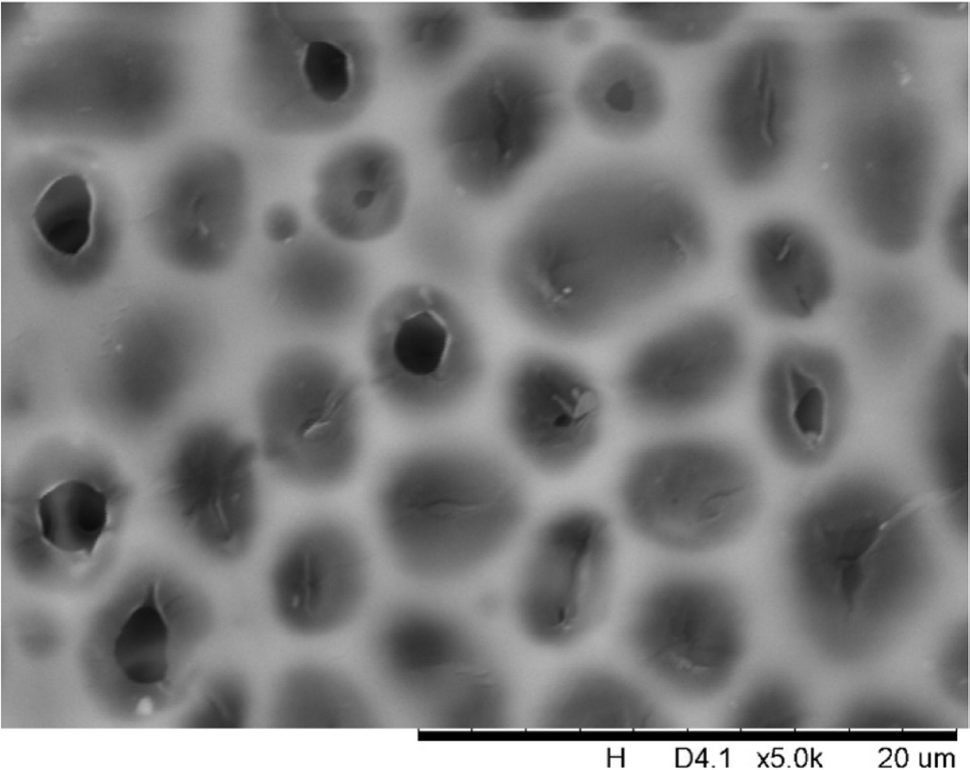
Surface of PES-PEG blend membrane, MMM-0.01F, prepared with 0.01 wt% of functionalised MWCNTs

**Fig. 31:**
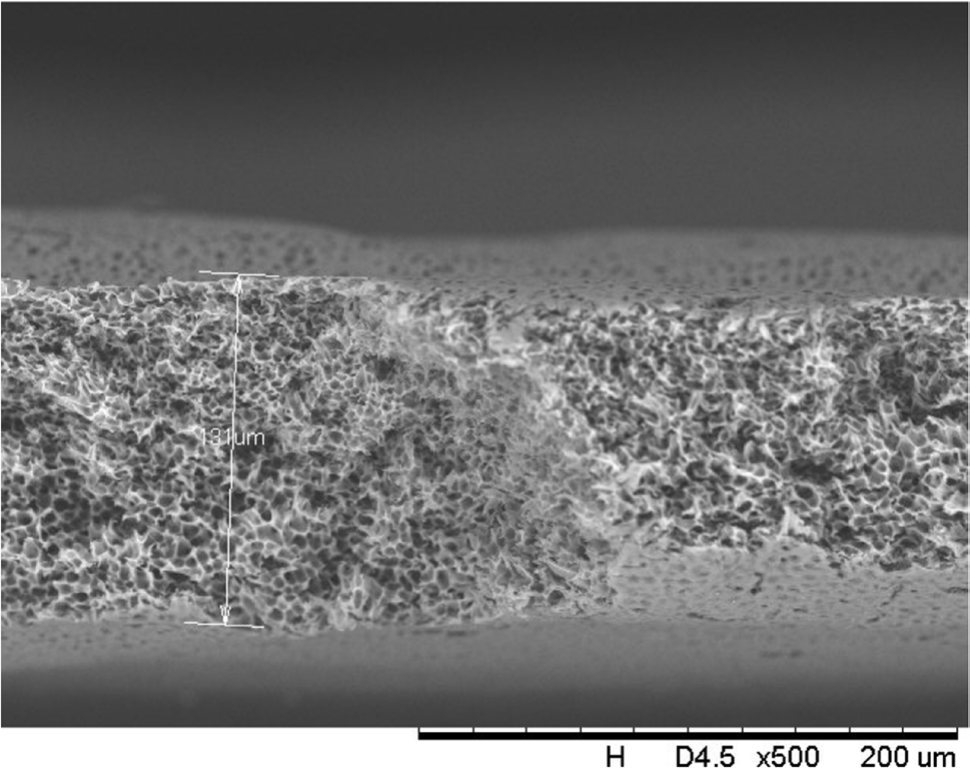
Cross section of PES-PEG blend membrane, MMM-0.02F, prepared with 0.02 wt% of functionalised MWCNTs

**Fig. 32:**
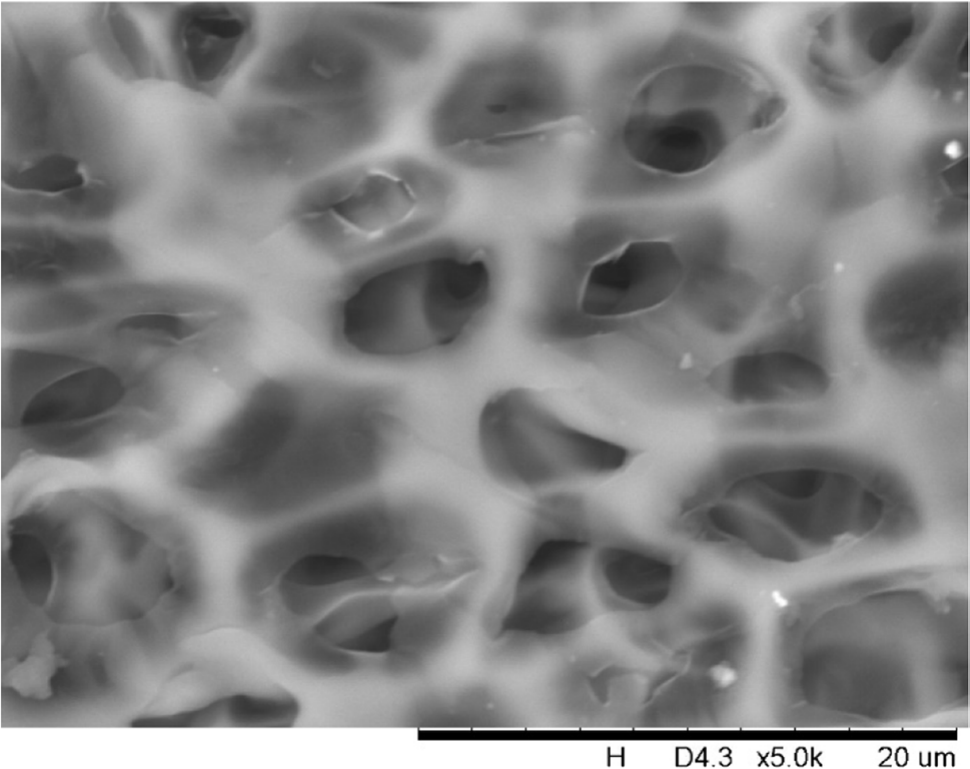
Surface of PES-PEG blend membrane, MMM-0.02F, prepared with 0.02 wt% of functionalised MWCNTs

**Fig. 33:**
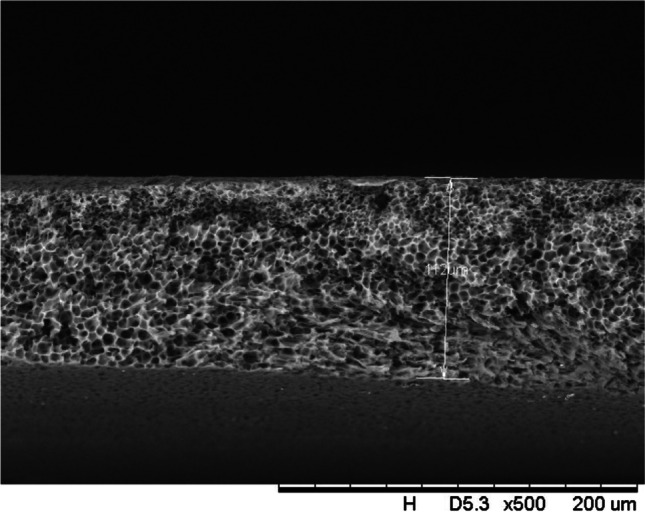
Cross section of PES-PEG blend membrane, MMM-0.03F, prepared with 0.03 wt% of functionalised MWCNTs

**Fig. 34:**
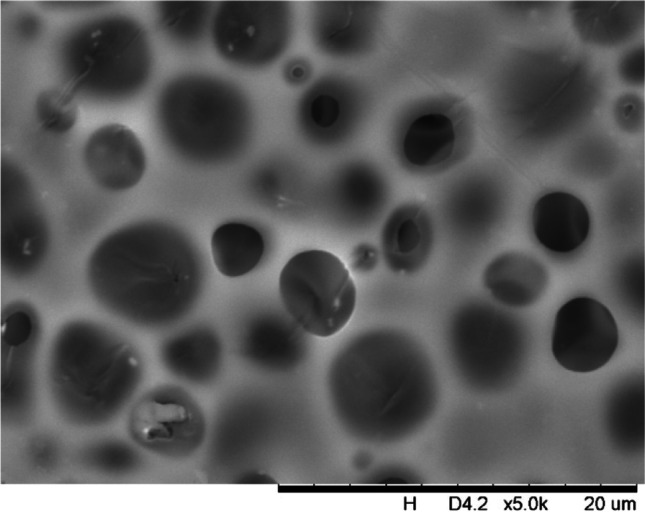
Surface of PES-PEG blend membrane, MMM-0.03F, prepared with 0.03 wt% of functionalised MWCNTs

**Fig. 35:**
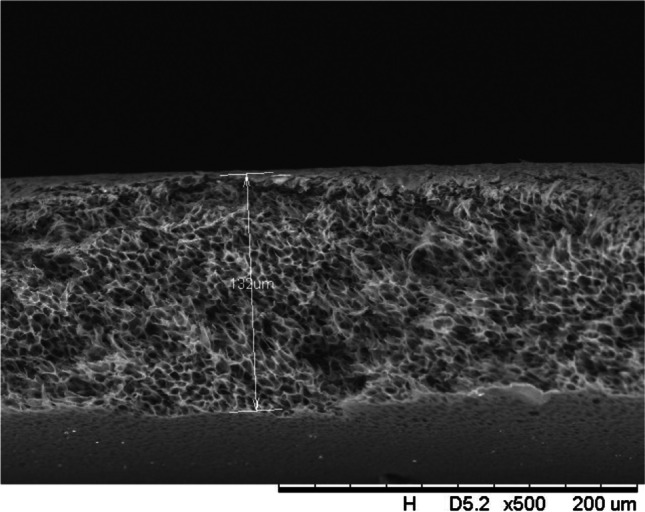
Cross section of PES-PEG blend membrane, MMM-0.035F, prepared with 0.035 wt% of functionalised MWCNTs

**Fig. 36:**
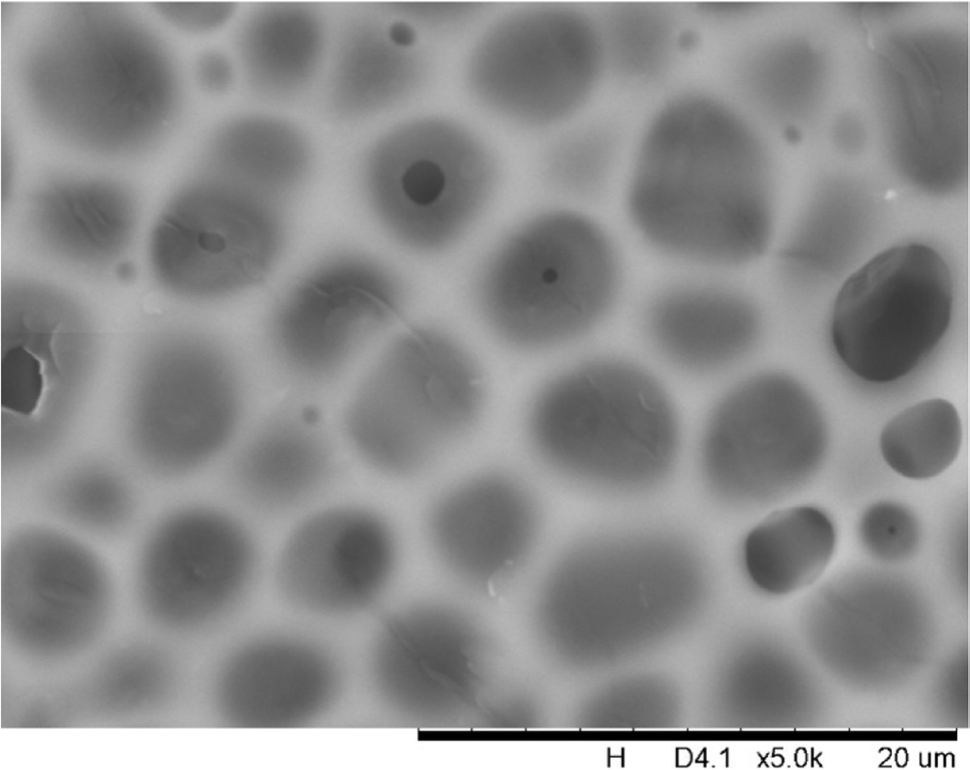
Surface of PES-PEG blend membrane, MMM-0.035F, prepared with 0.035 wt% of functionalised MWCNTs

**Fig. 37:**
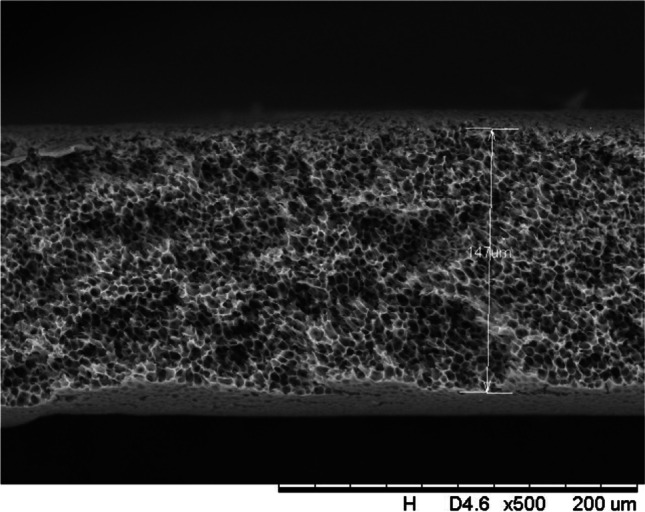
Cross section of PES-PEG blend membrane, MMM-0.04F, prepared with 0.04 wt% of functionalised MWCNTs

**Fig. 38:**
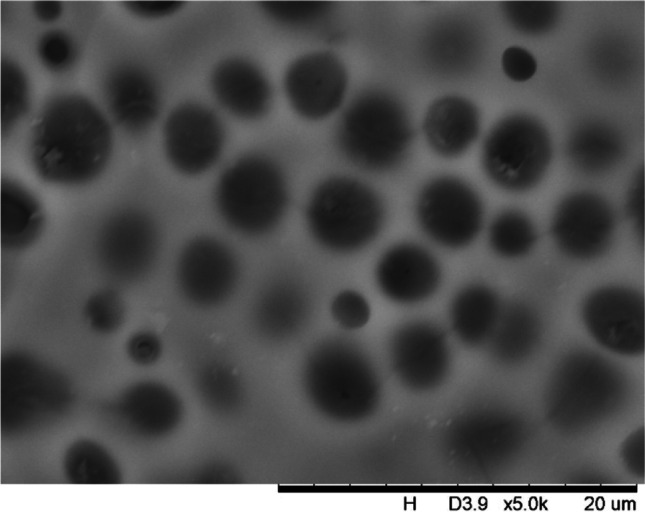
Surface of PES-PEG blend membrane, MMM-0.04F, prepared with 0.04 wt% of functionalised MWCNTs

**Fig. 39:**
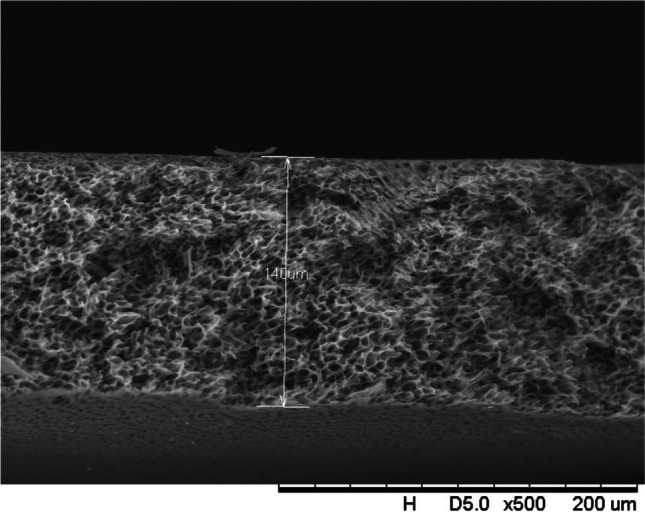
Cross section of PES-PEG blend membrane, MMM-0.05F, prepared with 0.05 wt% of functionalised MWCNTs

**Fig. 40:**
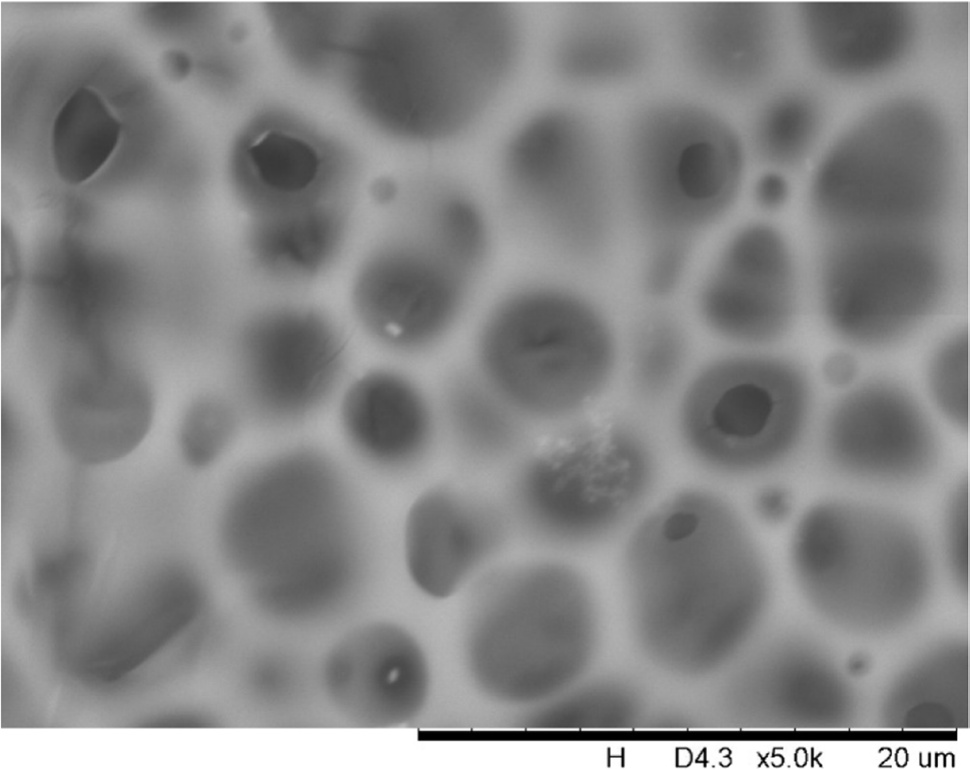
Surface of PES-PEG blend membrane, MMM-0.05F, prepared with 0.05 wt% of functionalised MWCNTs

In addition, the surface cross-sectional morphologies of the blend MMMs, MMM-0.01F, MMM-0.02F, MMM-0.03F, MMM-0.035F, MMM-0.04F and MMM-0.05F were investigated using SEM analysis (Figs. 29, 30, 31, 32, 33, 34, 35, 36, 37, 38, 39 and 40) to better understand the gas transportation behaviour of the membranes. Figures 29, 31, 33, 35, 37 and 39 show membranes with asymmetric and sponge-like structures throughout. Due to the increase in MWCNTs-F loadings from 0.01 to 0.02 wt%, an increase in the thickness from 158 to 163 μm is allowed. This could be due to the presence of hydrophilic β-CD that coalesces the polymer chains in the skin layer; resulting in a thicker and denser layer (Ahmad et al. 2014). In addition, the viscosity of the dope solution is significantly affected by the loading of functionalised MWCNTs which build up a denser membrane structure (A.L. Ahmad et al. 2014).

### MMM-0.03F operating pressures

#### CO_2_ and N_2_ separation performance

**Fig. 41:**
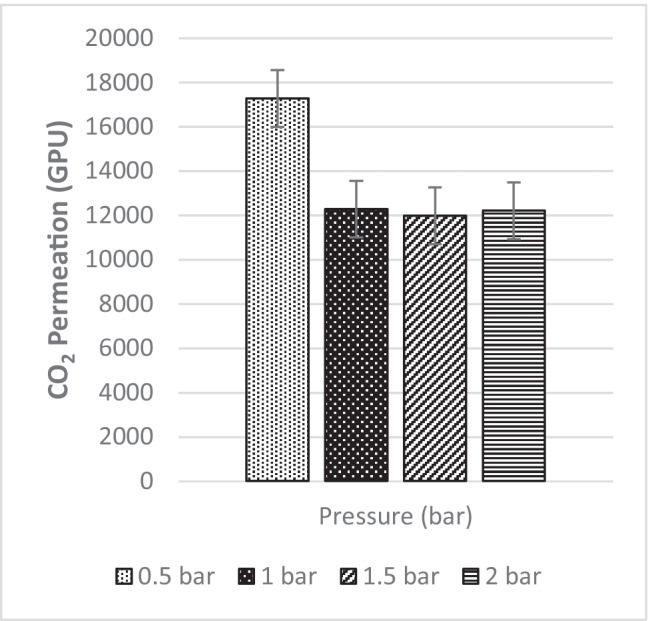
CO_2_ permeance of MMM-0.03F prepared with PES 20 wt%, PEG 20 wt%, NMP 29.81 wt%, DMF 29.81 wt% and MWCNTs-F 0.03 wt%

**Fig. 42:**
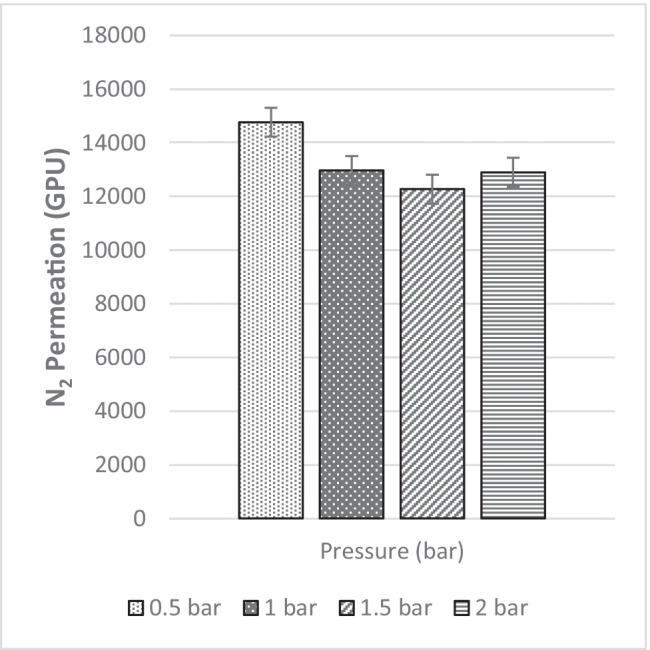
N_2_ permeance of MMM-0.03F prepared with PES 20 wt%, PEG 20 wt%, NMP 29.81 wt%, DMF 29.81 wt% and MWCNTs-F 0.03

In the following study, a single permeation test for CO_2_ (Fig. 41) and N_2_ (Fig. 42) was carried out on MMM-0.03F with different pressures to investigate the operating pressure. The readings obtained are 17,282.95 GPU, 12,284.81 GPU, 11,985.19 GPU and 12,211.72 GPU at 0.5 bar, 1 bar, 1.5 bar and 2 bar, respectively. In Fig. 41, a decrease in the CO_2_ permeance of MMM-0.03F from 0.5 to 1 bar was followed by a fluctuation between 1 and 2 bar. The reduction in permeance from 0.5 to 1 bar is expected due to the saturation of the Langmuir adsorption site (Zhang et al. 2019). On the other hand, the increase that is experienced after could be due to the presence of PEG, creating a rubbery polymer where pressure increases. Additionally, the increase in permeance could be due to the presence of PES, causing the sorption of condensable CO_2_ gas into the glassy polymer (Farnam et al. 2014). As a result, plasticisation occurs. However, the increase in CO_2_ permeance could be a combination of the two reasons mentioned above.

**Fig. 43:**
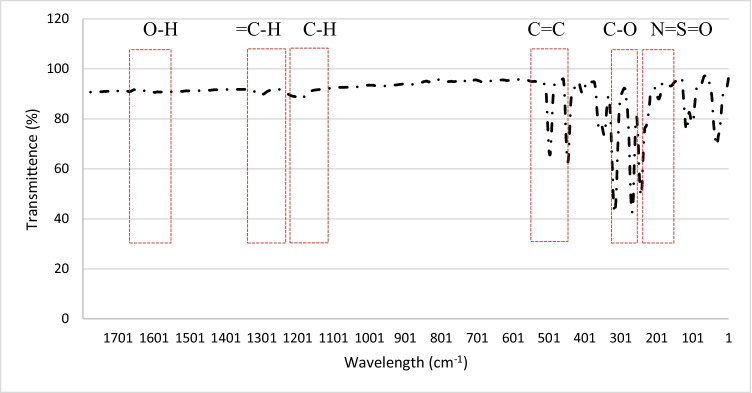
FTIR results of MMM-0.03F prepared with PES 20 wt%, PEG 20 wt%, NMP 29.81 wt%, DMF 29.81 wt% and MWCNTs-F 0.03 wt%

Furthermore, the FTIR results for MMM-0.03F (Fig. 43) indicate that the O–H stretching bond is the highest, resulting in a larger presence of O–H bonds compared to the previous membranes. With the higher O–H bonds, a better CO_2_ permeance results due to the stronger dipole-quadruple interaction between the polar O–H groups and the non-polar molecules.

**Fig. 44:**
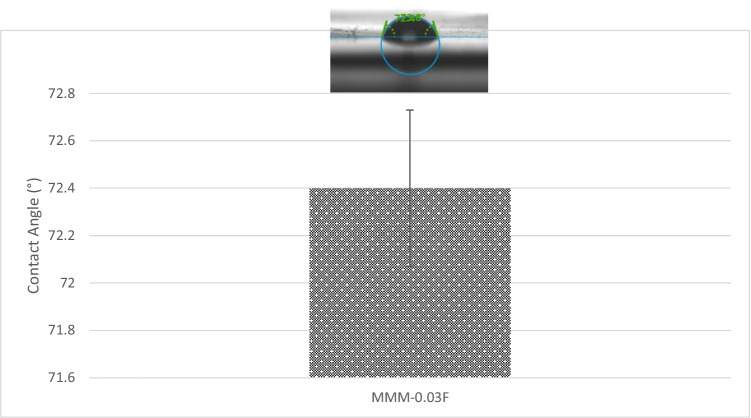
Contact angle results of MMM-0.03F prepared with PES 20 wt%, PEG 20 wt%, NMP 29.81 wt%, DMF 29.81 wt% and MWCNTs-F 0.03 wt%

The N_2_ permeance for MMM-0.03F is described in Fig. 42. At 0.5 bar (14,768.06 GPU) to 1.5 bar (12,274.42 GPU), the permeance of N_2_ decreases due to the saturation of Langmuir sorption sites, which causes the steep decrease from 0.5 to 1 bar. This could also be explained via hydrophilicity. This is illustrated in Fig. 44, which shows the contact angles of MMM-0.03F. An increase in hydrophilicity would mean that the C–O group would be larger in the polymer matrix (Lee et al. 2018). This trend is observed in the FTIR results of Fig. 43, where the peak of C–O is 1209.52 cm^−1^. Additionally, the FTIR result in Fig. 43 shows that there is a high absorbance of C–O, which increases the CO_2_ permeance and, thus, hinders the N_2_ permeance in the process. It could be a possible reason to the drop in N_2_ permeance. However, when pressure is increased from 1 bar (12,971.36 GPU) to 2 bar (12,903.38 GPU), the value starts to fluctuate around each other. This is due to Henry’s law-based solution transport starting to contribute to the gas permeance (Lasseuguette et al. 2018; M. Zhang et al. 2019).

**Fig. 45:**
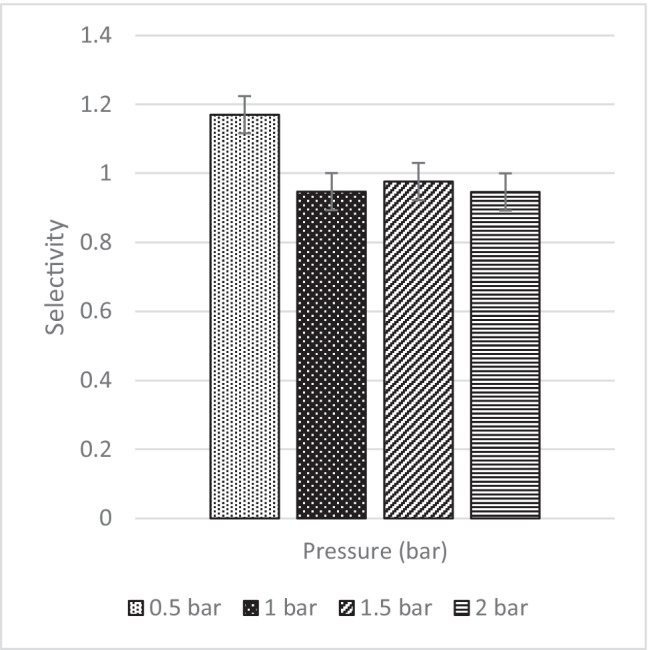
CO_2_/N_2_ Selectivity of MMM-0.03F prepared with PES 20 wt%, PEG 20 wt%, NMP 29.81 wt%, DMF 29.81 wt% and MWCNTs-F 0.03 wt%

The CO_2_/N_2_ selectivity is represented in Fig. 45 below at the four operating pressures. From the data collected, their respective selectivity is 1.17, 0.95, 0.98 and 0.95 at 0.5 bar, 1 bar, 1.5 bar and 2 bar, respectively. As seen in Fig. 41, when the CO_2_ pressure increases, plasticisation occurs at 0.5 bar. As observed in Fig. 45, upon plasticisation, the CO_2_ selectivity increases. Fortunately, MMM-0.03F is not plasticised due to good performance. The contact angle for MMM-0.03F is 72.4°, making it a strong hydrophilic surface. This can be further supported with the higher absorption peaks of C–O and O–H bonds in the polymer matrix which promotes CO_2_ permeance, resulting in a higher CO_2_ selectivity. Furthermore, based on Fig. 45, MMM-0.03F demonstrates a good performance within the industrial range (1 to 2 bar). This is explained by the PEG that adsorbs CO_2_ molecules due to the high affinity of polar ether bonds present. The increase in CO_2_ permeance is due to the increment in the rubbery structure of PEG, which in turn increases the solubility of the blend MMM.

## Conclusion

In conclusion, the integration of MWCNTs and the composition of PES/PEG in the blend mixed matrix membrane (MMM) play important roles in enhancing the gas separation performance of a membrane. From the Hansen solubility parameter (HSP) study and the experimental procedures carried out in the above sections, it was found that MWCNTs-F had a better gas separation property compared to MWCNTs-P. This was due to the MWCNTs-F having shorter and less agglomerate behaviour achieved from the Chen soft cutting method. Overall, the optimum MWCNTs-F loading was found to be 0.03 wt% for MMM-0.03F. At 0.03 wt% MWCNTs-F along with PES:PEG weight ratio of 20:20, this allowed for the highest selectivity to be achieved at 1.01 + 0.05 while achieving a CO_2_ and N_2_ permeance of 13,441.17 GPU and 13,229.31 GPU, respectively. The maximum operating pressure tested on MMM-0.03F was 2 bar. This indicates that MMM-0.03F has good mechanical strength due to its composition of polymers. Hence, this allows it to be suitable for post-combustion application in the industries where the typical flue gas pressure is at 1.2 bar.

## Data Availability

Readily available.
